# Supplementary material for: Sequence-Based Prediction of Protein Phase Separation: The Role of Beta-Pairing Propensity
Source: Biomolecules. 2022 Nov 28;12(12):1771. doi: 10.3390/biom12121771 (PMC9775558; doi:10.3390/biom12121771)
Supplement: Supplementary file 1 [file biomolecules-12-01771-s001.zip › fileS2.pdf]

Sequences from the LLPS positive set which are classified correctly by score s_4 and incorrectly by PScore, against the negative set hsnLLPS, with a false positive FPR=0.3 as the precision threshold (set $\tilde{S}_4$)

The sequence stretches selected by PASTA for the best β-pairing are highlighted in yellow background. All pairings are predicted to be in-register parallel. Sequences are ranked from the highest to the lowest value of the score s_4.

>sp|Q14978|NOLC1_HUMAN Nucleolar and coiled-body phosphoprotein 1 OS=Homo sapiens OX=9606 GN=NOLC1 PE=1 SV=2

MADAGIRRVVPSDLYPLVLGFLRDNQLSEVANKFAKATGATQQDANASSLLDIYSFWLKS
AKVPERKLQANGPVAKKAKKKASSSDSEDSSEEEEEVQGPPAKKAAVPAKRVGLPPGKAA
AKASESSSSEESSDDDDEEDQKKQPVQKGVKPQAKAAKAPPKKAKSSDSDSDSSSEDEPP
KNQKPKITPVTVKAQTKAPPKPARAAPKIANGKAASSSSSSSSSSSDDSEEEKAAATPK
KTVPKKQVVAKAPVKAATTPTRKSSSSEDSSSDEEEEQKKPMKNKPGPYSSVPPPSAPPP
KKSLGTQPPKKAVEKQQPVESSEDSSDESDSSSEEEKKPPTKAVVSKATTKPPPAKKAAE
SSSDSSDSDSSEDDEAPSKPAGTTKNSSNKPAVTTKSPAVKPAAAPKQPVGGGQKLLTRK
ADSSSSEEESSSSEEEKTKKMVATTKPKATAKAALSLPAKQAPQGSRDSSSDSDSSSSEE
EEEKTSKSAVKKKPQKVAGGAAPSKPASAKKGKAESSNSSSSDDSSEEEEEKLKGKGSPR
PQAPKANGTSALTAQNGKAAKNSEEEEEEEKKKAAVVVSKSGSLKKRKQNEAAKEAETPQA
KKIKLQTPNTFPKRKKGEKRASSPFRRVREEEIEVDSRVADNSFDAKRGAAGDWGERANQ
VLKFTKGKSFRHEKTKKKRGSYRGGSISVQVNSIKFDSE

>sp|O76064|RNF8_HUMAN E3 ubiquitin-protein ligase RNF8 OS=Homo sapiens OX=9606 GN=RNF8 PE=1 SV=1

MGEPGFFVTGDRAGGRSWCLRRVGMSAGWLLLEDGCEVTVGRGFGVTYQLVSKICPLMIS
RNHCVLKQNPEGQWTIMDNKSLNGVWLNRARLEPLRVYSIHQGDYIQLGVPLENKENAEY
EYEVTEEDWETIYPCLSPKNDQMIEKNKELRTKRKFSLDELAGPGAEGPSNLKSKINKVS
CESGQPVKSQGKGEVASTPSDNLDPKLTALEPSKTTGAPIYPGFPKVTEVHHEQKASNSS
ASQRSLQMFKVTMSRILRLKIQMQEKHEAVMNVKKQTQKGNSKKVVQMEQELQDLQSQLC
AEQAQQQARVEQLEKTFQEEEQHLQGLEIAQGEKDLKQQLAQALQEHWALMEELNRSKKD
FEAIIQAKNKELEQTKEEKEKMQAQKEEVLSHMNDVLENELQCIICSEYFIEAVTLNCAH
SFCSYCINEWMKRKIECPICRKDIKSKTYSLVLDNCINKMVNNLSSEVKERRIVLIRERK
AKRLF

>sp|O13828|DCP2_SCHPO mRNA decapping complex subunit 2 OS=Schizosaccharomyces pombe (strain 972 / ATCC 24843) OX=284812 GN=dcp2 PE=1 SV=1

MSFTNATFSQVLDDLSARFILNLPAEEQSSVERLCFQIEQAHWFYEDFIRAQNDQLPSLG
LRVFSAKLFAHCPLLWKWSKVHEEAFDDFLRYKTRIPVRGAIMLDMSMQQCVLVKGWKAS
SGWGFPKGKIDKDESDVDCAIREVYEETGFDCSSRINPNEFIDMTIRGQNVRLYIIPGIS
LDTRFESRTRKEISKIEWHNLMDLPTFKKNKPQTMKNKFYMVIPFLAPLKKWIKKRNIAN
NTTKEKNISVDVDADASSQLLSLLKSSTAPSDLATPQPSTFPQPPVESHSSFDIKQKILH
LLNEGNEPKSPIQLPPVSNLPLNPPIQSSNSRLSHDNNSFDPFAYLGLDPKNPSASFPRV
VSQNNMLTNKPVLNNHFQQSMYSNLLKDQNSVQHLFAASDMPSPMELPSPSTVYHQVFYP
PTSTSVSSYGLGKTPQPAYGSSSPYVNGHQTQQISSLPPFQSQTQFLARNSDNSGQSYNS
EGDSNSKRLLSMLSQQDTTPSSSTLSKEANVQLANLFLTPNSLETKKFSDNSQGEEISDN
LHGESCNNPNANSVHSAQLLQALLHPSATETKEETPKKTSDSLSLLTLLKSGLPTPANDL
QNKSQNNERKASSQVKELEVKNYSKSTDLLKKTLRIPRNDEPLEAANQFDLLKVSPQQKS
EVPPKRNELSQSKLKNRKKKENSETNKNHVDMSPGFVKILKRSPLADQKKEDTQESDFKG
SDDHFLSYLQSVVSSNSNGLH

>sp|P11940|PABP1_HUMAN Polyadenylate-binding protein 1 OS=Homo sapiens OX=9606 GN=PABPC1 PE=1 SV=2

MNPSAPSYPMASLYVGDLHPDVTEAMLYEKFSPAGPILSIRVCRDMITRRSLGYAYVNFQ
QPADAERALDTMNFDVIKGKPVRIMWSQRDPSLRKSGVGNIFIKNLDKSIDNKALYDTFS
AFGNILSCKVVCDENGSKGYGFVHFETQEAAERAIEKMNGMLLNDRKVFVGRFKSRKERE
AELGARAKEFTNVYIKNFGEDMDDERLKDLFGKFGPALSVKVMTDESGKSKGFGFVSFER
HEDAQKAVDEMNGKELNGKQIYVGRAQKKVERQTELKRKFEQMKQDRITRYQGVNLYVKN
LDDGIDDERLRKEFSPFGTITSAKVMMEGGRSKGFGFVCFSSPEEATKAVTEMNGRIVAT
KPLYVALAQRKEERQAHLTNQYMQRMASVRAVPNPVINPYQPAPPSGYFMAAIPQTQNRA
AYYPPSQIAQLRPSPRWTAQGARPHPFQNMPGAIRPAAPRPPFSTMRPASSQVPRVMSTQ
RVANTSTQTMGPRPAAAAAAATPAVRTVPQYKYAAGVRNPQQHLNAQPQVTMQQPAVHVQ
GQEPLTASMLASAPPQEQKQMLGERLFPLIQAMHPTLAGKITGMLLEIDNSELLHMLESP
ESLRSKVDEAVAVLQAHQAKEAAQKAVNSATGVPTV

>sp|P20028|RPA2_DROME DNA-directed RNA polymerase I subunit RPA2 OS=Drosophila melanogaster OX=7227 GN=RpI135 PE=1 SV=2

MLEEMQQMKTIPVLTNSRPEFKQIPKKLSRHLANLGGPHVDSFDEMLTVGLDNSAKHMIP
NHWLSPAGEKISMKVESIWIAKPKVPQDVIDVRTREIYPTDSRQLHVSYSGMCSVRLGWS
VNGVQKTPINMDLGEVPIMLRSKACNLGQATPEEMVKHGEHDSEWGGIFVIRGNEKIVRM
LIMTRRNHPICVKRSSWKDRGQNFSDLGMLVQTVREDESSLSNVVHYLNNGTAKFMFSHV
KRLSYVPVCLILKCLMDYTDEEIYNRLVQGYESDQYYVSCVQAMLREVQNENVYTHAQCK

SFIGNLFRARFPEVPEWQPDDDVTDFILRERVMIHLDTYEDKFQLIVFMIQKLFQCAQGK
YKVENVDSAMMQEVLLPGHLYQKYLSERVESWVSQVRRCLQKKLTSPDALVTSAVMTQCM
RQAGGVGRAIESFLATGNIASRTGLGLMQNSGLVIMAENINRMRYMSHFRAIHRGSYFTT
MRTTEARQLLPDAWGFICPVHTPDGTPCGLLNHLTLTCEISMRPDPKLVKAIPKHLIDMG
MMPLSNRRYLGEKLYVVFLDGKHLGHIHQSEAEKIVDELRYGKIFGTLPQMMEIGFIPFK
KNGQFPGLYIATGPARMMRPVWNLKWKRVEYIGTLEQLYMEIAIDAKEMYPDFTTHLELA
KTHFMSNLANLIPMPDYNQSPRNMYQCQMGKQTMGTPCLNWPKQAANKLYRLQTPGTPLF
RPVHYDNIQLDDFAMGTNAIVAVISYTGYDMEDAMIINKAAYERGFAYGSIYKTKFLTLD
KKSSYFARHPHMPELIKHLDTDGLPHPGSKLSYGSPLYCYFDGEVATYKVVKMDEKEDCI
VESIRQLGSFDLSPKKMVAITLRVPRPATIGDKFASRAGQKGICSQKYPAEDLPFTESGL
IPDIVFNPHGFPSRMTIAMMIETMAGKGAAIHGNVYDATPFRFSEENTAIDYFGKMLEAG
GYNYYGTERLYSGVDGREMTADIFFGVVHYQRLRHMVFDKWQVRSTGAVEARTHQPIKGR
KRGGGVRFGEMERDALISHGAAFLLQDRLFHNSDKTHTLVCHKCGSILAPLQRIVKRNET
GGLSSQPDTCRLCGDNSSVSMIEIPFSFKYLVTELSSVNINARFKLNEI

>sp|P35974|PHOSP_MEASA Phosphoprotein OS=Measles virus (strain Edmonston-AIK-C vaccine) OX=36408 GN=P/V PE=1 SV=1

MAEEQARHVKNGLECIRALKAEPIGSLAIEEAMAAWSEISDNPGQERATCREEKAGSSGL
SKPCLSAIGSTEGGAPRIRGQGPGESDDDAETLGIPPRNLQASSTGLQCYYVYDHSGEAV
KGIQDADSIMVQSGLDGDSTLSGGDNESENSDVDIGEPDTEGYAITDRGSAPISMGFRAS
DVETAEGGEIHELLRLQSRGNNFPKLGKTLNVPPPPDPGRASTSGTPIKKGTERRLASFG
TEIASLLTGGATQCARKSPSEPSGPGAPAGNVPEYVSNAALIQEWTPESGTTISPRSQNN
EEGGDYYDDELFSDVQDIKTALAKIHEDNQKIISKLESLLLLKGEVESIKKQINRQNISI
STLEGHLSSIMIAIPGLGKDPNDPTADVEINPDLKPIIGRDSGRALAEVLKKPVASRQLQ
GMTNGRTSSRGQLLKEFQPKPIGKKMSSAVGFVPDTGPASRSVIRSIIKSSRLEEDRKRY
LMTLLDDIKGANDLAKFHQMLMKIIMK

>sp|Q86YR5|GPSM1_HUMAN G-protein-signaling modulator 1 OS=Homo sapiens OX=9606 GN=GPSM1 PE=1 SV=2

MAGPAPPVADELPGPAARRLYSRMEASCLELALEGERLCKAGDFKTGVAFFEAAVQVGTE
DLKTLSAIYSQLGNAYFYLKEHGRALEYHKHDLLLARTIGDRMGEAKASGNLGNTLKVLG
RFDEAAVCCQRHLSIAQEQGDKVGEARALYNIGNVYHAKGKQLSWNAANATQDPGHLPPD
VRETLCKASEFYERNLSLVKELGDRAAQGRAYGNLGNTHYLLGNFTEATTFHKERLAIAK
EFGDKAAERRAYSNLGNAHVFLGRFDVAAEYYKKTLQLSRQLRDQAVEAQACYSLGNTYT
LLQDYERAAEYHLRHLLIAQELADRVGEGRACWSLGNAYVSMGRPAQALTFAKKHLQISQ
EIGDRHGELTARMNVAQLQLVLGRLTSPAASEKPDLAGYEAQGARPKRTQRLSAETWDLL
RLPLEREQNGDSHHSGDWRGPSRDSLPLPVRSRKYQEGPDAERRPREGSHSPLDSADVRV
HVPRTSIPRAPSSDEECFFDLLTKFQSSRMDDQRCPLDDGQAGAAEATAAPTLEDRIAQP
SMTASPQTEEFFDLIASSQSRRLDDQRASVGSLPGLRITHSNAGHLRGHGEPQEPGDDFF

NMLIKYQSSRIDDQRCPPPDVLPRGPTMPDEDFFSLIQRVQAKRMDEQRVDLAGGPEQGA
GGPPEPQQQCQPGAS

>sp|P11387|TOP1_HUMAN DNA topoisomerase 1 OS=Homo sapiens OX=9606 GN=TOP1 PE=1 SV=2

MSGDHLHNDSQIEADFRLNDSHKHKDKHKDREHRHKEHKKEKDREKSKHSNSEHKDSEKK
HKEKEKTKHKDGSSEKHKDKHKDRDKEKRKEEKVRASGDAKIKKEKENGFSSPPQIKDEP
EDDGYFVPPKEDIKPLKRPRDEDDADYKPKKIKTEDTKKEKKRKLEEEEDGKLKKPKNKD
KDKKVPEPDNKKKKPKKEEEQKWKWWEEERYPEGIKWKFLEHKGPVFAPPYEPLPENVKF
YYDGKVMKLSPKAEEVATFFAKMLDHEYTTKEIFRKNFFKDWRKEMTNEEKNIITNLSKC
DFTQMSQYFKAQTEARKQMSKEEKLKIKEENEKLLKEYGFCIMDNHKERIANFKIEPPGL
FRGRGNHPKMGMLKRRIMPEDIIINCSKDAKVPSPPPGHKWKEVRHDNKVTWLVSWTENI
QGSIKYIMLNPSSRIKGEKDWQKYETARRLKKCVDKIRNQYREDWKSKEMKVRQRAVALY
FIDKLALRAGNEKEEGETADTVGCCSLRVEHINLHPELDGQEYVVEFDFLGKDSIRYYNK
VPVEKRVFKNLQLFMENKQPEDDLFDRLNTGILNKHLQDLMEGLTAKVFRTYNASITLQQ
QLKELTAPDENIPAKILSYNRANRAVAILCNHQRAPPKTFEKSMMNLQTKIDAKKEQLAD
ARRDLKSAKADAKVMKDAKTKKVVESKKKAVQRLEEQLMKLEVQATDREENKQIALGTSK
LNYLDPRITVAWCKKWGVPIEKIYNKTQREKFAWAIDMADEDYEF

>sp|Q9Y6X2|PIAS3_HUMAN E3 SUMO-protein ligase PIAS3 OS=Homo sapiens OX=9606 GN=PIAS3 PE=1 SV=2

MAELGELKHMVMSFRVSELQVLLGFAGRNKSGRKHELLAKALHLLKSSCAPSVQMKIKEL
YRRRFPRKTLGPSDLSLLSLPPGTSPVGSPGPLAPIPPTLLAPGTLLGPKREVDMHPPLP
QPVHPDVTMKPLPFYEVYGELIRPTTLASTSSQRFEEAHFTFALTPQQVQQILTSREVLP
GAKCDYTIQVQLRFCLCETSCPQEDYFPPNLFVKVNGKLCPLPGYLPPTKNGAEPKRPSR
PINITPLARLSATVPNTIVVNWSSEFGRNYSLSVYLVRQLTAGTLLQKLRAKGIRNPDHS
RALIKEKLTADPDSEVATTSLRVSLMCPLGKMRLTVPCRALTCAHLQSFDAALYLQMNEK
KPTWTCPVCDKKAPYESLIIDGLFMEILSSCSDCDEIQFMEDGSWCPMKPKKEASEVCPP
PGYGLDGLQYSPVQGGDPSENKKKVEVIDLTIESSSDEEDLPPTKKHCSVTSAAIPALPG
SKGVLTSGHQPSSVLRSPAMGTLGGDFLSSLPLHEYPPAFPLGADIQGLDLFSFLQTESQ
HYGPSVITSLDEQDALGHFFQYRGTPSHFLGPLAPTLGSSHCSATPAPPGRVSSIVAPG
GALREGHGGPLPSGPSLTGCRSDIISLD

>sp|Q9UKV8|AGO2_HUMAN Protein argonaute-2 OS=Homo sapiens OX=9606 GN=AGO2 PE=1 SV=3

MYSGAGPALAPPAPPPPIQGYAFKPPPRPDFGTSGRTIKLQANFFEMDIPKIDIYHYELD

IKPEKCPRRVNREIVEHMVQHFKTQIFGDRKPVFDGRKNLYTAMPLPIGRDKVELEVTLP
GEGKDRIFKVSIKWVSCVSLQALHDALSGRLPSVPFETIQALDVVMRHLPSMRYTPVGRS
FFTASEGCSNPLGGGREVWFGFHQSVRPSLWKMMLNIDVSATAFYKAQPVIEFVCEVLDF
KSIEEQQKPLTDSQRVKFTKEIKGLKVEITHCGQMKRKYRVCNVTRRPASHQTFPLQQES
GQTVECTVAQYFKDRHKLVLRYPHLPCLQVGQEQKHTYLPLEVCNIVAGQRCIKKLTDNQ
TSTMIRATARSAPDRQEEISKLMRSASFNTDPYVREFGIMVKDEMTDVTGRVLQPPSILY
GGRNKAIATPVQGVWDMRNKQFHTGIEIKVWAIACFAPQRQCTEVHLKSFTEQLRKISRD
AGMPIQGQPCFCKYAQGADSVEPMFRHLKNTYAGLQLVVVILPGKTPVYAEVKRVGDTVL
GMATQCVQMKNVQRTTPQTLSNLCLKINVKLGGVNNILLPQGRPPVFQQPVIFLGADVTH
PPAGDGKKPSIAAVVGSMDAHPNRYCATVRVQQHRQEIIQDLAAMVRELLIQFYKSTRFK
PTRIIFYRDGVSEGQFQQVLHHELLAIREACIKLEKDYQPGITFIVVQKRHHTRLFCTDK
NERVGKSGNIPAGTTVDTKITHPTEFDFYLCSHAGIQGTSRPSHYHVLWDDNRFSSDELQ
ILTYQLCHTYVRCTRSVSIPAPAYYAHLVAFRARYHLVDKEHDSAEGSHTSGQSNGRDHQ
ALAKAVQVHQDTLRTMYFA

>sp|Q9H9Y6|RPA2_HUMAN DNA-directed RNA polymerase I subunit RPA2 OS=Homo sapiens OX=9606 GN=POLR1B PE=1 SV=2

MDPGSRWRNLPSGPSLKHLTDPSYGIPREQQKAALQELTRAHVESFNYAVHEGLGLAVQA
IPPFEFAFKDERISFTILDAVISPPTVPKGTICKEANVYPAECRGRRSTYRGKLTADINW
AVNGISKGIIKQFLGYVPIMVKSKLCNLRNLPPQALIEHHEEAEEMGGYFIINGIEKVIR
MLIMPRRNFPIAMIRPKWKTRGPGYTQYGVSMHCVREEHSAVNMNLHYLENGTVMLNFIY
RKELFFLPLGFALKALVSFSDYQIFQELIKGKEDDSFLRNSVSQMLRIVMEEGCSTQKQV
LNYLGECFRVKLNVPDWYPNEQAAEFLFNQCICIHLKSNTEKFYMLCLMTRKLFALAKGE
CMEDNPDSLVNQEVLTPGQLFLMFLKEKLEGWLVSIKIAFDKKAQKTSVSMNTDNLMRIF
TMGIDLTKPFEYLFATGNLRSKTGLGLLQDSGLCVVADKLNFIRYLSHFRCVHRGADFAK
MRTTTVRRLLPESWGFLCPVHTPDGEPCGLMNHLTAVCEVVTQFVYTASIPALLCNLGVT
PIDGAPHRSYSECYPVLLDGVMVGWVDKDLAPGIADSLRHFKVLREKRIPPWMEVVLIPM
TGKPSLYPGLFLFTTPCRLVRPVQNLALGKEELIGTMEQIFMNVAIFEDEVFAGVTTHQE
LFPHSLLSVIANFIPFSDHNQSPRNMYQCQMGKQTMGFPLLTYQDRSDNKLYRLQTPQSP
LVRPSMYDYYDMDNYPIGTNAIVAVISYTGYDMEDAMIVNKASWERGFAHGSVYKSEFID
LSEKIKQGDSSLVFGIKPGDPRVLQKLDDDGLPFIGAKLQYGDPYYSYLNLNTGESFVMY
YKSKENCVVDNIKVCSNDTGSGKFKCVCITMRVPRNPTIGDKFASRHGQKGILSRLWPAE
DMPFTESGMVPDILFNPHGFPSRMTIGMLIESMAGKSAALHGLCHDATPFIFSEENSALE
YFGEMLKAAGYNFYGTERLYSGISGLELEADIFIGVVYYQRLRHMVSDKFQVRTTGARDR
VTNQPIGGRNVQGGIRFGEMERDALLAHGTSFLLHDRLFNCSDRSVAHVCVKCGSLLSPL
LEKPPPSWSAMRNRKYNCTLCSRSDTIDTVSVPYVFRYFVAELAAMNIKVKLDVV

>tr|B1MUE9|B1MUE9_DISMA Gamma M8c crystallin OS=Dissostichus mawsoni OX=36200 PE=2 SV=1

MSNTGMNMRGKITFYEEKNFQGRSYECMNDCSDMSSSLSRSQSCRVESGCFMVYDRNNYM
GNQYFMKRGEYSDYMSMMGMRDCIKSCRMIPMHRGQFRMKIYEKENFDGQSHDMMEDCDN
IMDRYRMNECQSCNVMDGHWLMYEQPQFRGKMMYMRPGEYRNFKDMGMSCQRFMSMRRIT
DSC

>sp|P17480|UBF1_HUMAN Nucleolar transcription factor 1 OS=Homo sapiens OX=9606 GN=UBTF PE=1 SV=1

MNGEADCPTDLEMAAPKGQDRWSQEDMLTLLECMKNNLPSNDSSKFKTTESHMDWEKVAF
KDFSGDMCKLKWVEISNEVRKFRTLTELILDAQEHVKNPYKGKKLKKHPDFPKKPLTPYF
RFFMEKRAKYAKLHPEMSNLDLTKILSKKYKELPEKKKMKYIQDFQREKQEFERNLARFR
EDHPDLIQNAKKSDIPEKPKTPQQLWYTHEKKVYLKVRPDATTKEVKDSLGKQWSQLSDK
KRLKWIHKALEQRKEYEEIMRDYIQKHPELNISEEGITKSTLTKAERQLKDKFDGRPTKP
PPNSYSLYCAELMANMKDVPSTERMVLCSQQWKLLSQKEKDAYHKKCDQKKKDYEVELLR
FLESLPEEEQQRVLGEEKMLNINKKQATSPASKKPAQEGGKGGSEKPKRPVSAMFIFSEE
KRRQLQEERPELSESELTRLLARMWNDLSEKKKAKYKAREAALKAQSERKPGGEREERGK
LPESPKRAEEIWQQSVIGDYLARFKNDRVKALKAMEMTWNNMEKKEKLMWIKKAAEDQKR
YERELSEMRAPPAATNSSKKMKFQGEPKKPPMNGYQKFSQELLSNGELNHLPLKERMVEI
GSRWQRISQSQKEHYKKLAEEQQKQYKVHLDLWVKSLSPQDRAAYKEYISNKRKSMTKLR
GPNPKSSRTTLQSKSESEEDDEEDEDDEDEDEEEEDDENGDSSEDGGDSSESSSEDESED
GDENEEDDEDEDDDEDDDEDEDNESEGSSSSSSSSGDSSDSDSN

>sp|O75683|SURF6_HUMAN Surfeit locus protein 6 OS=Homo sapiens OX=9606 GN=SURF6 PE=1 SV=3

MASLLAKDAYLQSLAKKICSHSAPEQQARTRAGKTQGSETAGPPKKKRKKTQKKFRKREE
KAAEHKAKSLGEKSPAASGARRPEAAKEEAAWASSSAGNPADGLATEPESVFALDVLRQR
LHEKIQEARGQGSAKELSPAALEKRRRRKQERDRKKRKRKELRAKEKARKAEEATEAQEV
VEATPEGACTEPREPPGLIFNKVEVSEDEPASKAQRRKEKRQRVKGNLTPLTGRNYRQLL
ERLQARQSRLDELRGQDEGKAQELEAKMKWTNLLYKAEGVKIRDDERLLQEALKRKEKRR
AQRQRRWEKRTAGVVEKMQQRQDRRRQNLRRKKAARAERRLLRARKKGRILPQDLERAGL
V

>tr|Q9VUU4|Q9VUU4_DROME Decapping protein 2, isoform A OS=Drosophila melanogaster OX=7227 GN=DCP2 PE=3 SV=4

MEIAPLINNAVAVVTASASAAVSASASVGSSSKDDNVNNLALLASLTLQKVLNINNNNNQ

HSKNNNHSDNSNSKQHEQQKQQQQPQRRNLDIKSEYDVLNQLLKTGAVFTATPTAPIAIE
RRKQTQQQQRPYAQQAHQQQQQKQRLNSSISSTHSSTSTNSSYGSSSSEDAAGTSAPTGA
GEVVTSAALTPRASTTKASSNKLPEKSKIPSDILDDLASRFIINVPDMELNNLIRMCFQI
ELAHWFYLDFFCAPESGEDGETPKCVQRKLPSVGIKQFAMQLFQHIPFLNKHFGTVDQIL
DEWKNYKLSVPTYGAILVSEDHNHCLLVQSYFARNSWGFPKGKINENEDPAHCATREVYE
ETGFDITDLIDANDYIEAFINYQYTRLYVVRNIPMDTQFAPRTRNEIKCCDWFRIDALPV
NKNDAISKAKLGKTSNSFFMIMPFVKRLKKWVNDRKAGIEPRRRKFSGQQSPKAASPTNM
NGNCKKDVTGVRNESQRQRHKSMGDLDGVKLNNLNGKGTVGTGAGSAASASATAAINSMN
ICNSNGKRNKQNAAAGSNQATPTTIATPMTSNGAVVGGTVSSKRQLFHSQSQNDAQQSAS
NEKIVSSFDLIRKEKQQQLKAAKAQKQQQQQQQRSRTKSQSDKQYNPQQPVKILGQQQPT
TGMDLIAMLSAAAAAQKTPNNEQQTQEKQQQQQRPRPASVSLNLGAGSATGATTSAPDAQ
YMHRLQRMASGTNLRILKRAQSQQPQQHHQQQLQLEQQQQQQQQLGIDFTPNLNSWTNFS
FTKNFIANVFC

>sp|Q01826|SATB1_HUMAN DNA-binding protein SATB1 OS=Homo sapiens OX=9606 GN=SATB1 PE=1 SV=1

MDHLNEATQGKEHSEMSNNVSDPKGPPAKIARLEQNGSPLGRGRLGSTGAKMQGVPLKHS
GHLMKTNLRKGTMLPVFCVVEHYENAIEYDCKEEHAEFVLVRKDMLFNQLIEMALLSLGY
SHSSAAQAKGLIQVGKWNPVPLSYVTDAPDATVADMLQDVYHVVTLKIQLHSCPKLEDLP
PEQWSHTTVRNALKDLLKDMNQSSLAKECPLSQSMISSIVNSTYYANVSAAKCQEFGRWY
KHFKKTKDMMVEMDSLSELSQQGANHVNFGQQPVPGNTAEQPPSPAQLSHGSQPSVRTPL
PNLHPGLVSTPISPQLVNQQLVMAQLLNQQYAVNRLLAQQSLNQQYLNHPPPVSRSMNKP
LEQQVSTNTEVSSEIYQWVRDELKRAGISQAVFARVAFNRTQGLLSEILRKEEDPKTASQ
SLLVNLRAMQNFLQLPEAERDRIYQDERERSLNAASAMGPAPLISTPPSRPPQVKTATIA
TERNGKPENNTMNINASIYDEIQQEMKRAKVSQALFAKVAATKSQGWLCELLRWKEDPSP
ENRTLWENLSMIRRFLSLPQPERDAIYEQESNAVHHHGDRPPHIIHVPAEQIQQQQQQQQ
QQQQQQQAPPPPQPQQQPQTGPRLPPRQPTVASPAESDEENRQKTRPRTKISVEALGILQ
SFIQDVGLYPDEEAIQTLSAQLDLPKYTIIKFFQNQRYYLKHHGKLKDNSGLEVDVAEYK
EEELLKDLEESVQDKNTNTLFSVKLEEELSVEGNTDINTDLKD

>sp|O62275|WAGO4_CAEEL Argonaute protein wago-4 OS=Caenorhabditis elegans OX=6239 GN=wago-4 PE=1 SV=1

MPALPPVYTPSGAPSSVHAPPAVPPVPVPTQPLRSEYQTSNDACIKRLEELNIAPAAKLY
PTPTEPGKCGVEAEIQTNVFGIEMHQDSLFYQYSVNITTELKNGKEVTFTKKGKDDFVVT
ERHDKCCAILFRALGDYEEFFKTSDSCLIYDGQSILFSNVDLFQGFREGAVKTKYMQLDG
GEMDHKDLKSLPCIKLEVFPTKNPAVKFTREAVARRATDSNLDSVSLAYQQILELALTQP
CLRNTARYVVFDHGKMFFIDPLGEGFEKCDVVDVGDGKQVVPGLKKTINFIEGPYGRGRS
NPSVVIDGMKVAFHKNQPILDKLKEITTQPVEHGLKGLEKDRCAAVIKGLDCYSTYGGRE
RHHKIEGIHHEGARNARFELNDGGSCTVAQYFEDVYNITLRYPDTNLIVSKERGNINFYP

MELLKISSHQRVQIPQLTSAQSQKTTKESAVLPDVRQRLILTGKNAAQISSDNEVLGKMG
VSVCEDPLMVKGRSIPAVKLANAEIGANPINVKDNKWRANRFTRPATAPNVWAMYVVGTA
STRITLDTLKKFADEFAAMCKSKGVNMPAPADISLIHMDAIESRLYDATKANCTFVFIIT
DDSITTLHQRYKMIEKDTKMIVQDMKLSKALSVINAGKRLTLENVINKTNVKLGGSNYVF
VDAKKQLDSHLIIGVGISAPPAGTKYAMENKGVLNPNVIGYAYNAQHNQEFSGDFVLNSA
SQDTLAPIEDIVMHSLNEYQKFHDGGLPRRVIVYRTGTSEGNHGSIMAYEIPLARAAMRD
FSPDIQLVYIVVSKDHSFRFFKPDLASLASRPQATSSTASRHSAMPAAPKAWDLNIAPGI
LVDSIVTNPACKQFFLNSHITLQGTAKTPLYTVLADDAKVSMTALEDITYKLCHLHQIVG
LPTSLPTPLYVANEYAKRGRNLWNEAVALNNVPTVSGPEADRLKELTKSICYKASGDLTG
RRVNA

>sp|P54132|BLM_HUMAN Bloom syndrome protein OS=Homo sapiens OX=9606 GN=BLM PE=1 SV=1

MAAVPQNNLQEQLERHSARTLNNKLSLSKPKFSGFTFKKKTSSDNNVSVTNVSVAKTPVL
RNKDVNVTEDFSFSEPLPNTTNQQRVKDFFKNAPAGQETQRGGSKSLLPDFLQTPKEVVC
TTQNTPTVKKSRDTALKKLEFSSSPDSLSTINDWDDMDDFDTSETSKSFVTPPQSHFVRV
STAQKSKKGKRNFFKAQLYTTNTVKTDLPPPSSESEQIDLTEEQKDDSEWLSSDVICIDD
GPIAEVHINEDAQESDSLKTHLEDERDNSEKKKNLEEAELHSTEKVPCIEFDDDDYDTDF
VPPSPEEIISASSSSSKCLSTLKDLDTSDRKEDVLSTSKDLLSKPEKMSMQELNPETSTD
CDARQISLQQQLIHVMEHICKLIDTIPDDKLKLLDCGNELLQQRNIRRKLLTEVDFNKSD
ASLLGSLWRYRPDSLDGPMEGDSCPTGNSMKELNFSHLPSNSVSPGDCLLTTTLGKTGFS
ATRKNLFERPLFNTHLQKSFVSSNWAETPRLGKKNESSYFPGNVLTSTAVKDQNKHTASI
NDLERETQPSYDIDNFDIDDFDDDDDWEDIMHNLAASKSSTAAYQPIKEGRPIKSVSERL
SSAKTDCLPVSSTAQNINFSESIQNYTDKSAQNLASRNLKHERFQSLSFPHTKEMMKIFH
KKFGLHNFRTNQLEAINAALLGEDCFILMPTGGGKSLCYQLPACVSPGVTVVISPLRSLI
VDQVQKLTSLDIPATYLTGDKTDSEATNIYLQLSKKDPIIKLLYVTPEKICASNRLISTL
ENLYERKLLARFVIDEAHCVSQWGHDFRQDYKRMNMLRQKFPSVPVMALTATANPRVQKD
ILTQLKILRPQVFSMSFNRHNLKYYVLPKKPKKVAFDCLEWIRKHHPYDSGIIYCLSRRE
CDTMADTLQRDGLAALAYHAGLSDSARDEVQQKWINQDGCQVICATIAFGMGIDKPDVRF
VIHASLPKSVEGYYQESGRAGRDGEISHCLLFYTYHDVTRLKRLIMMEKDGNHHTRETHF
NNLYSMVHYCENITECRRIQLLAYFGENGFNPDFCKKHPDVSCDNCCKTKDYKTRDVTDD
VKSIVRFVQEHSSSQGMRNIKHVGPSGRFTMNMLVDIFLGSKSAKIQSGIFGKGSAYSRH
NAERLFKKLILDKILDEDLYINANDQAIAYVMLGNKAQTVLNGNLKVDFMETENSSSVKK
QKALVAKVSQREEMVKKCLGELTEVCKSLGKVFGVHYFNIFNTVTLKKLAESLSSDPEVL
LQIDGVTEDKLEKYGAEVISVLQKYSEWTSPAEDSSPGISLSSSRGPGRSAAEELDEEIP
VSSHYFASKTRNERKRKKMPASQRSKRRKTASSGSKAKGGSATCRKISSKTKSSSIIGSS
SASHTSQATSGANSKLGIMAPPKPINRPFLKPSYAFS

>sp|Q9BY44|EIF2A_HUMAN Eukaryotic translation initiation factor 2A OS=Homo sapiens OX=9606 GN=EIF2A PE=1 SV=3

MAPSTPLLTVRGSEGLYMVNGPPHFTESTVFPRESGKNCKVCIFSKDGTLFAWGNGEKVN
IISVTNKGLLHSFDLLKAVCLEFSPKNTVLATWQPYTTSKDGTAGIPNLQLYDVKTGTCL
KSFIQKKMQNWCPSWSEDETLCARNVNNEVHFFENNNFNTIANKLHLQKINDFVLSPGPQ
PYKVAVYVPGSKGAPSFVRLYQYPNFAGPHAALANKSFFKADKVTMLWNKKATAVLVIAS
TDVDKTGASYYGEQTLHYIATNGESAVVQLPKNGPIYDVVWNSSSTEFCAVYGFMPAKAT
IFNLKCDPVFDFGTGPRNAAYYSPHGHILVLAGFGNLRGQMEVWDVKNYKLISKPVASDS
TYFAWCPDGEHILTATCAPRLRVNNGYKIWHYTGSILHKYDVPSNAELWQVSWQPFLDGI
FPAKTITYQAVPSEVPNEEPKVATAYRPPALRNKPITNSKLHEEEPPQNMKPQSGNDKPL
SKTALKNQRKHEAKKAAKQEARSDKSPDLAPTPAPQSTPRNTVSQSISGDPEIDKKIKNL
KKKLKAIEQLKEQAATGKQLEKNQLEKIQKETALLQELEDLELGI

>sp|Q9BZC1|CELF4_HUMAN CUGBP Elav-like family member 4 OS=Homo sapiens OX=9606 GN=CELF4 PE=1 SV=1

MYIKMATLANGQADNASLSTNGLGSSPGSAGHMNGLSHSPGNPSTIPMKDHDAIKLFIGQ
IPRNLDEKDLKPLFEEFGKIYELTVLKDRFTGMHKGCAFLTYCERESALKAQSALHEQKT
LPGMNRPIQVKPADSESRGGSSCLRQPPSQDRKLFVGMLNKQQSEDDVRRLFEAFGNIEE
CTILRGPDGNSKGCAFVKYSSHAEAQAAINALHGSQTMPGASSSLVVKFADTDKERTMRR
MQQMAGQMGMFNPMAIPFGAYGAYAQALMQQQAALMASVAQGGYLNPMAAFAAAQMQQMA
ALNMNGLAAAPMTPTSGGSTPPGITAPAVPSIPSPIGVNGFTGLPPQANGQPAAEAVFAN
GIHPYPAQSPTAADPLQQAYAGVQQYAGPAAYPAAYGQISQAFPQPPPMIPQQQREGPEG
CNLFIYHLPQEFGDAELMQMFLPFGFVSFDNPASAQTAIQAMNGFQIGMKRLKVQLKRPK
DANRPY

>sp|Q14191|WRN_HUMAN Werner syndrome ATP-dependent helicase OS=Homo sapiens OX=9606 GN=WRN PE=1 SV=2

MSEKKLETTAQQRKCPEWMNVQNKRCAVEERKACVRKSVFEDDLPFLEFTGSIVYSYDAS
DCSFLSEDISMSLSDGDVVGFDMEWPPLYNRGKLGKVALIQLCVSESKCYLFHVSSMSVF
PQGLKMLLENKAVKKAGVGIEGDQWKLLRDFDIKLKNFVELTDVANKKLKCTETWSLNSL
VKHLLGKQLLKDKSIRCSNWSKFPLTEDQKLYAATDAYAGFIIYRNLEILDDTVQRFAIN
KEEEILLSDMNKQLTSISEEVMDLAKHLPHAFSKLENPRRVSILLKDISENLYSLRRMII
GSTNIETELRPSNNLNLLSFEDSTTGGVQQKQIREHEVLIHVEDETWDPTLDHLAKHDGE
DVLGNKVERKEDGFEDGVEDNKLKENMERACLMSLDITEHELQILEQQSQEEYLSDIAYK
STEHLSPNDNENDTSYVIESDEDLEMEMLKHLSPNDNENDTSYVIESDEDLEMEMLKSLE
NLNSGTVEPTHSKCLKMERNLGLPTKEEEEDDENEANEGEEDDDKDFLWPAPNEEQVTCL
KMYFGHSSFKPVQWKVIHSVLEERRDNVAVMATGYGKSLCFQYPPVYVGKIGLVISPLIS
LMEDQVLQLKMSNIPACFLGSAQSENVLTDIKLGKYRIVYVTPEYCSGNMGLLQQLEADI
GITLIAVDEAHCISEWGHDFRDSFRKLGSLKTALPMVPIVALTATASSSIREDIVRCLNL
RNPQITCTGFDRPNLYLEVRRKTGNILQDLQPFLVKTSSHWEFEGPTIIYCPSRKMTQQV

TGELRKLNLSCGTYHAGMSFSTRKDIHHRFVRDEIQCVIATIAFGMGINKADIRQVIHYG
APKDMESYYQEIGRAGRDGLQSSCHVLWAPADINLNRHLLTEIRNEKFRLYKLKMMAKME
KYLHSSRCRRQIILSHFEDKQVQKASLGIMGTEKCCDNCRSRLDHCYSMDDSEDTSWDFG
PQAFKLLSAVDILGEKFGIGLPILFLRGSNSQRLADQYRRHSLFGTGKDQTESWWKAFSR
QLITEGFLVEVSRYNKFMKICALTKKGRNWLHKANTESQSLILQANEELCPKKLLLPSSK
TVSSGTKEHCYNQVPVELSTEKKSNLEKLYSYKPCDKISSGSNISKKSIMVQSPEKAYSS
SQPVISAQEQETQIVLYGKLVEARQKHANKMDVPPAILATNKILVDMAKMRPTTVENVKR
IDGVSEGKAAMLAPLLEVIKHFCQTNSVQTDLFSSTKPQEEQKTSLVAKNKICTLSQSMA
ITYSLFQEKKMPLKSIAESRILPLMTIGMHLSQAVKAGCPLDLERAGLTPEVQKIIADVI
RNPPVNSDMSKISLIRMLVPENIDTYLIHMAIEILKHGPDSGLQPSCDVNKRRCFPGSEE
ICSSSKRSKEEVGINTETSSAERKRRLPVWFAKGSDTSKKLMDKTKRGGLFS

>sp|P25655|NOT1_YEAST General negative regulator of transcription subunit 1 OS=Saccharomyces cerevisiae (strain ATCC 204508 / S288c) OX=559292 GN=CDC39 PE=1 SV=3

MLSATYRDLNTASNLETSKEKQAAQIVIAQISLLFTTLNNDNFESVEREIRHILDRSSVD
IYIKVWERLLTLSSRDILQAGKFLLQENLLHRLLLEFAKDLPKKSTDLIELLKERTFNNQ
EFQKQTGITLSLFIDLFDKSANKDIIESLDRSSQINDFKTIKMNHTNYLRNFFLQTTPET
LESNLRDLLHSLEGESLNDLLALLLSEILSPGSQNLQNDPTRSWLTPPMVLDATNRGNVI
ARSISSLQANQINWNRVFNLMSTKYFLSAPLMPTTASLSCLFAALHDGPVIDEFFSCDWK
VIFKLDLAIQLHKWSVQNGCFDLLNAEGTRKVSETIPNTKQSLLYLLSIASLNLELFLQR
EELSDGPMLAYFQECFFEDFNYAPEYLILALVKEMKRFVLLIENRTVIDEILITLLIQVH
NKSPSSFKDVISTITDDSKIVDAAKIIINSDDAPIANFLKSLLDTGRLDTVINKLPFNEA
FKILPCARQIGWEGFDTFLKTKVSPSNVDVVLESLEVQTKMTDTNTPFRSLKTFDLFAFH
SLIEVLNKCPLDVLQLQRFESLEFSLLIAFPRLINFGFGHDEAILANGDIAGINNDIEKE
MQNYLQKMYSGELAIKDVIELLRRLRDSDLPRDQEVFTCITHAVIAESTFFQDYPLDALA
TTSVLFGSMILFQLLRGFVLDVAFRIIMRFAKEPPESKMFKFAVQAIYAFRIRLAEYPQY
CKDLLRDVPALKSQAQVYQSIVEAATLANAPKERSRPVQEMIPLKFFAVDEVSCQINQEG
APKDVVEKVLFVLNNVTLANLNNKVDELKKSLTPNYFSWFSTYLVTQRAKTEPNYHDLYS
KVIVAMGSGLLHQFMVNVTLRQLFVLLSTKDEQAIDKKHLKNLASWLGCITLALNKPIKH
KNIAFREMLIEAYKENRLEIVVPFVTKILQRASESKIFKPPNPWTVGILKLLIELNEKAN
WKLSLTFEVEVLLKSFNLTTKSLKPSNFINTPEVIETLSGALGSITLEQQQTEQQRQIIL
MQQHQQQMLIYQQRQQQQQQRQQQQHHISANTIADQQAAFGGEGSISHDNPFNNLLGST
IFVTHPDLKRVFQMALAKSVREILLEVVEKSSGIAVVTTTKIILKDFATEVDESKLKTAA
IIMVRHLAQSLARATSIEPLKEGIRSTMQSLAPNLMSLSSSPAEELDTAINENIGIALVL
IEKASMDKSTQDLADQLMQAIAIRRYHKERRADQPFITQNTNPYSLSLPEPLGLKNTGVT
PQQFRVYEEFGKNIPNLDVIPFAGLPAHAPPMTQNVGLTQPQQQQAQMPTQILTSEQIRA
QQQQQQLQKSRLNQPSQSAQPPGVNVPNPQGGIAAVQSDLEQNQRVLVHLMDILVSQIKE
NATKNNLAELGDQNQIKTIIFQILTFIAKSAQKDQLALKVSQAVVNSLFATSESPLCREV
LSLLLEKLCSLSLVARKDVVWWLVYALDSRKFNVPVIRSLLEVNLIDATELDNVLVTAMK
NKMENSTEFAMKLIQNTVLSDDPILMRMDFIKTLEHLASSEDENVKKFIKEFEDTKIMPV
RKGTKTTRTEKLYLVFTEWVKLLQRVENNDVITTVFIKQLVEKGVISDTDNLLTFVKSSL
ELSVSSFKESDPTDEVFIAIDALGSLIIKLLILQGFKDDTRRDYINAIFSVIVLVFAKDH
SQEGTTFNERPYFRLFSNILYEWATIRTHNFVRISDSSTRQELIEFDSVFYNTFSGYLHA
LQPFAFPGFSFAWVTLLSHRMLLPIMLRLPNKIGWEKLMLLIIDLFKFLDQYTSKHAVSD

AVSVVYKGTLRVILGISNDMPSFLIENHYELMNNLPPTYFQLKNVILSAIPKNMTVPNPY
DVDLNMEDIPACKELPEVFFDPVIDLHSLKKPVDNYLRIPSNSLLRTILSAIYKDTYDIK
KGVGYDFLSVDSKLIRAIVLHVGIEAGIEYKRTSSNAVFNTKSSYYTLLFNLIQNGSIEM
KYQIILSIVEQLRYPNIHTYWFSFVLMNMFKSDEWNDQKLEVQEIILRNFLKRIIVNKPH
TWGVSVFFTQLINNNDINLLDLPFVQSVPEIKLILQQLVKYSKKYTTSEQDDQSATINRR
QTPLQSNA

>sp|Q500V5|AGDP1_ARATH Protein AGENET DOMAIN (AGD)-CONTAINING P1 OS=Arabidopsis thaliana OX=3702 GN=AGDP1 PE=1 SV=1

MLRPRRSLGVSSPAKQRKKAAPKNSMATRANRKRLPSYLKPGSAVEISSDEIGFRGSWYM
GKVITIPSSSDKDSVKCQVEYTTLFFDKEGTKPLKEVVDMSQLRPPAPPMSEIEKKKKIV
VGEEVDAFYNDGWWEGDVTEVLDDGKFSVFFRSSKEQIRFRKDELRFHREWVDGAWKPPL
EETEEEEDESEEDKLDDSEDEEDILARVDLETTRAIAKQMFSSGTVVEVSSDEEGFQGCW
FAAKVVEPVGEDKFLVEYRDLREKDGIEPLKEETDFLHIRPPPPRDEDIDFAVGDKINAF
YNDGWWVGVVIDGMKHGTVGIYFRQSQEKMRFGRQGLRLHKDWVDGTWQLPLKGGKIKRE
KTVSCNRNVRPKKATEKQAFSIGTPIEVSPEEEGFEDSWFLAKLIEYRGKDKCLVEYDNL
KAEDGKEPLREEVNVSRIRPLPLESVMVSPFERHDKVNALYNDGWWVGVIRKVLAKSSYL
VLFKNTQELLKFHHSQLRLHQEWIDGKWITSFKSQKV

>tr|B1MUE1|B1MUE1_DISMA Gamma M1 crystallin OS=Dissostichus mawsoni OX=36200 PE=2 SV=1

MGKIVFYEEKNFQGRSYECMSDCSDMSSYLSRCQSCRVESGCFMTYERPNYMGNQFFMRK
GEYQDMQRMMSMGMMFDTIRSCRMIPFHRGQFRMRIHEKENFGGQMNELMDDCDNIQERY
RMNECQSCNVMEGHWLMYEQPQFRGKMMYMRPGEYRNFKDMGMSGQRFMSMRRITDMC

>sp|Q9VD51|DDX18_DROME Probable ATP-dependent RNA helicase pitchoune OS=Drosophila melanogaster OX=7227 GN=pit PE=2 SV=2

MSIREKLLMKKIVKREKMKKELSQKKGNKNAQKQEPPKQNGNKPSKKPEKLSKKHVAKDE
DDDLEEDFQEAPLPKKKQQKQPPKKQQIQVANSDSESDDDEQEDEADEDSDLDEVAEVDE
EDVDSGSEDDDQQEDEDEEEPVPAKKTKLLPNKSKAQNGKPAKDDEPFTVESSLAALDYR
DSDDRSFASLKGAVSEATLRAIKEMGFTEMTEIQSKSLTPLLKGRDLVGAAQTGSGKTLA
FLIPAVELINKLRFMPRNGTGVIIISPTRELSMQTFGVLKELMAHHHHTYGLVMGGSNRQ
VESEKLGKGINILVATPGRLLDHLQNSPDFLYKNLQCLIIDEVDRILEIGFEEELKQIIN
LLPKRRQTMLFSATQTARIEALSKLALKSEPIYVGVHDNQDTATVDGLEQGYIVCPSEKR
LLVLFTFLKKNRKKKVMVFFSSCMSVKYHHELFNYIDLPVTSIHGKQKQTKRTTTFFQFC

NAESGILLCTDVAARGLDIPQVDWIVQYDPPDDPREYIHRVGRTARGSGTSGHALLLMRP
EELGFLRYLKAAKVPLNEFEFSWQKIADIQLQLEKLIAKNYFLNQSAKEAFKSYVRAYDS
HQLKQIFNVNTLDLQAVAKSFGFLVPPVVDLKVGAAKRERPEKRVGGGGFGFYKKMNEGS
ASKQRHFKQVNRDQAKKFMR

>tr|B1MUE8|B1MUE8_DISMA Gamma M8b crystallin OS=Dissostichus mawsoni OX=36200 PE=2 SV=1

MSNTDMNMRGKIIFYEEKNFQGRSYECMNDCSDISSYLSRCQSVRVESGCFMVYDRNNYM
GNQYFMRRGEYSDYMSMMGMRENIKSCRMIPMHRGQFRMKIYERENFGGQSHEMMDDCEN
IQERYRMSDCQSCNVMDGHWLMYEQPNFRGKMMYMRPGEYRSFREMGMSGIKFMSMKRIT
DSCY

>sp|Q13283|G3BP1_HUMAN Ras GTPase-activating protein-binding protein 1 OS=Homo sapiens OX=9606 GN=G3BP1 PE=1 SV=1

MVMEKPSPLLVGREFVRQYYTLLNQAPDMLHRFYGKNSSYVHGGLDSNGKPADAVYGQKE
IHRKVMSQNFTNCHTKIRHVDAHATLNDGVVVQVMGLLSNNNQALRRFMQTFVLAPEGSV
ANKFYVHNDIFRYQDEVFGGFVTEPQEESEEEVEEPEERQQTPEVVPDDSGTFYDQAVVS
NDMEEHLEEPVAEPEPDPEPEPEQEPVSEIQEEKPEPVLEETAPEDAQKSSSPAPADIAQ
TVQEDLRTFSWASVTSKNLPPSGAVPVTGIPPHVVKVPASQPRPESKPESQIPPQRPQRD
QRVREQRINIPPQRGPRPIREAGEQGDIEPRRMVRHPDSHQLFIGNLPHEVDKSELKDFF
QSYGNVVELRINSGGKLPNFGFVVFDDSEPVQKVLSNRPIMFRGEVRLNVEEKKTRAARE
GDRRDNRLRGPGGPRGGLGGGMRGPPRGGMVQKPGFGVGRGLAPRQ

>sp|Q15717|ELAV1_HUMAN ELAV-like protein 1 OS=Homo sapiens OX=9606 GN=ELAVL1 PE=1 SV=2

MSNGYEDHMAEDCRGDIGRTNLIVNYLPQNMTQDELRSLFSSIGEVESAKLIRDKVAGHS
LGYGFVNYVTAKDAERAINTLNGLRLQSKTIKVSYARPSSEVIKDANLYISGLPRTMTQK
DVEDMFSRFGRIINSRVLVDQTTGLSRGVAFIRFDKRSEAEEAITSFNGHKPPGSSEPIT
VKFAANPNQNKNVALLSQLYHSPARRFGGPVHHQAQRFRFSPMGVDHMSGLSGVNVPGNA
SSGWCIFIYNLGQDADEGILWQMFGPFGAVTNVKVIRDFNTNKCKGFGFVTMTNYEEAAM
AIASLNGYRLGDKILQVSFKTNKSHK

>tr|Q9VNF8|Q9VNF8_DROME Protein transport protein SEC23 OS=Drosophila melanogaster OX=7227 GN=Sec23 PE=1 SV=2

MTTYEEFIQQNEDRDGVRLTWNVWPSSRIEASRLVVPLACLYQPLKERPDLPPIQYEPVL
CTRSNCRAILNPLCQVDYRAKLWVCNFCFQRNPFPPQYAAISEQHQPAELIPGFSTIEYT
ITRAPTMPPVFIFLVDTCMDEEELDALKDSLQMSLSLLPTNALVGLITFGKMIQVHELGA
EGCSKSYVFRGTKDLTAKQVQDMLGIGRGAAPGPQQQQHLPGQPAGAAAPVPPAHRFLQP
IGQCDAALGDLLSELQRDPWPVPQGKRYLRSTGAALSIAVGLLECTYPNTGGRIMTFVGG
PCSQGPGQVVDDELKHPIRSHHDIHKDNVRFMKKAIKHYDALALRAATNGHSVDIYSCAL
DQTGLLEMKQLCNSTGGHMVMGDSFNSSLFKQTFQRVFARDGRNDLKMAFNATLEVKCSR
ELKISGGIGSCVSLNVKSPSVSDVEIGMGNTVQWKLCTLNPSSTVAYFFEVVNQHAAPIP
QGGRGCIQFITQYQHPSGQRRIRVTTLARNWADATSNVHHISAGFDQEAAAVLMARMVVY
RAETDEGPDILRWVDRQLIRLCQKFGEYSKDDPNSFRLSQNFSLFPQFMYHLRRSQFLQV
FNNSPDETTFYRHMLMREDLTQSLIMIQPILYSYSFNGPPEPVLLDTASIQADRILLMDT
FFQILIYHGETIAQWRALKYQDMPEYENFKQLLQAPVDDAQEILQTRFPMPRYIDTEHGG
SQARFLLSKVNPSQTHNNMYAYGQDGGAPVLTDDVSLQLFMEHLKKLAVSTTT

>sp|Q03513|CCMM_SYNE7 Carboxysome assembly protein CcmM OS=Synechococcus elongatus (strain PCC 7942 / FACHB-805) OX=1140 GN=ccmM PE=1 SV=1

MPSPTTVPVATAGRLAEPYIDPAAQVHAIASIIGDVRIAAGVRVAAGVSIRADEGAPFQV
GKESILQEGAVIHGLEYGRVLGDDQADYSVWIGQRVAITHKALIHGPAYLGDDCFVGFRS
TVFNARVGAGSVIMMHALVQDVEIPPGRYVPSGAIITTQQQADRLPEVRPEDREFARHII
GSPPVIVRSTPAATADFHSTPTPSPLRPSSSEATTVSAYNGQGRLSSEVITQVRSLLNQG
YRIGTEHADKRRFRTSSWQPCAPIQSTNERQVLSELENCLSEHEGEYVRLLGIDTNTRSR
VFEALIQRPDGSVPESLGSQPVAVASGGGRQSSYASVSGNLSAEVVNKVRNLLAQGYRIG
TEHADKRRFRTSSWQSCAPIQSSNERQVLAELENCLSEHEGEYVRLLGIDTASRSRVFEA
LIQDPQGPVGSAKAAAAPVSSATPSSHSYTSNGSSSSDVAGQVRGLLAQGYRISAEVADK
RRFQTSSWQSLPALSGQSEATVLPALESILQEHKGKYVRLIGIDPAARRRVAELLIQKP

>sp|P03069|GCN4_YEAST General control protein GCN4 OS=Saccharomyces cerevisiae (strain ATCC 204508 / S288c) OX=559292 GN=GCN4 PE=1 SV=1

MSEYQPSLFALNPMGFSPLDGSKSTNENVSASTSTAKPMVGQLIFDKFIKTEEDPIIKQD
TPSNLDFDFALPQTATAPDAKTVLPIPELDDAVVESFFSSSTDSTPMFEYENLEDNSKEW
TSLFDNDIPVTTDDVSLADKAIESTEEVSLVPSNLEVSTTSFLPTPVLEDAKLTQTRKVK
KPNSVVKKSHHVGKDDESRLDHLGVVAYNRKQRSIPLSPIVPESSDPAALKRARNTEAAR
RSRARKLQRMKQLEDKVEELLSKNYHLENEVARLKKLVGER

>sp|P0DMV8|HS71A_HUMAN Heat shock 70 kDa protein 1A OS=Homo sapiens OX=9606 GN=HSPA1A PE=1 SV=1

MAKAAAIGIDLGTTYSCVGVFQHGKVEIIANDQGNRTTPSYVAFTDTERLIGDAAKNQVA
LNPQNTVFDAKRLIGRKFGDPVVQSDMKHWPFQVINDGDKPKVQVSYKGETKAFYPEEIS
SMVLTKMKEIAEAYLGYPVTNAVITVPAYFNDSQRQATKDAGVIAGLNVLRIINEPTAAA
IAYGLDRTGKGERNVLIFDLGGGTFDVSILTIDDGIFEVKATAGDTHLGGEDFDNRLVNH
FVEEFKRKHKKDISQNKRAVRRLRTACERAKRTLSSSTQASLEIDSLFEGIDFYTSITRA
RFEELCSDLFRSTLEPVEKALRDAKLDKAQIHDLVLVGGSTRIPKVQKLLQDFFNGRDLN
KSINPDEAVAYGAAVQAAILMGDKSENVQDLLLLDVAPLSLGLETAGGVMTALIKRNSTI
PTKQTQIFTTYSDNQPGVLIQVYEGERAMTKDNNLLGRFELSGIPPAPRGVPQIEVTFDI
DANGILNVTATDKSTGKANKITITNDKGRLSKEEIERMVQEAEKYKAEDEVQRERVSAKN
ALESYAFNMKSAVEDEGLKGKISEADKKKVLDKCQEVISWLDANTLAEKDEFEHKRKELE
QVCNPIISGLYQGAGGPGPGGFGAQGPKGGSGSGPTIEEVD

>sp|Q9BQ04|RBM4B_HUMAN RNA-binding protein 4B OS=Homo sapiens OX=9606 GN=RBM4B PE=1 SV=1

MVKLFIGNLPREATEQEIRSLFEQYGKVLECDIIKNYGFVHIEDKTAAEDAIRNLHHYKL
HGVNINVEASKNKSKASTKLHVGNISPTCTNQELRAKFEEYGPVIECDIVKDYAFVHMER
AEDAVEAIRGLDNTEFQGKRMHVQLSTSRLRTAPGMGDQSGCYRCGKEGHWSKECPVDRT
GRVADFTEQYNEQYGAVRTPYTMGYGESMYYNDAYGALDYYKRYRVRSYEAVAAAAAASA
YNYAEQTMSHLPQVQSTTVTSHLNSTSVDPYDRHLLPNSGAAATSAAMAAAAATTSSYYG
RDRSPLRRAAAMLPTVGEGYGYGPESELSQASAATRNSLYDMARYEREQYVDRARYSAF

>sp|Q9NZI8|IF2B1_HUMAN Insulin-like growth factor 2 mRNA-binding protein 1 OS=Homo sapiens OX=9606 GN=IGF2BP1 PE=1 SV=2

MNKLYIGNLNESVTPADLEKVFAEHKISYSGQFLVKSGYAFVDCPDEHWAMKAIETFSGK
VELQGKRLEIEHSVPKKQRSRKIQIRNIPPQLRWEVLDSLLAQYGTVENCEQVNTESETA
VVNVTYSNREQTRQAIMKLNGHQLENHALKVSYIPDEQIAQGPENGRRGGFGSRGQPRQG
SPVAAGAPAKQQQVDIPLRLLVPTQYVGAIIGKEGATIRNITKQTQSKIDVHRKENAGAA
EKAISVHSTPEGCSSACKMILEIMHKEAKDTKTADEVPLKILAHNNFVGRLIGKEGRNLK
KVEQDTETKITISSLQDLTLYNPERTITVKGAIENCCRAEQEIMKKVREAYENDVAAMSL
QSHLIPGLNLAAVGLFPASSSAVPPPPSSVTGAAPYSSFMQAPEQEMVQVFIPAQAVGAI
IGKKGQHIKQLSRFASASIKIAPPETPDSKVRMVIITGPPEAQFKAQGRIYGKLKEENFF
GPKEEVKLETHIRVPASAAGRVIGKGGKTVNELQNLTAAEVVVPRDQTPDENDQVIVKII

GHFYASQMAQRKIRDILAQVKQQHQKGQSNQAQARRK

>sp|O13892|YE38_SCHPO Uncharacterized protein C20G4.08 OS=Schizosaccharomyces pombe (strain 972 / ATCC 24843) OX=284812 GN=SPAC20G4.08 PE=1 SV=2

MNEQDLLNSLRRDLNLPNLGKSHDGSEAVESTFPEKKESSLSAQQPHVDDQRSSLLSLLN
AGLNASNQSPSNSGPKYYASHSSSTDALLQAFRDGAKPSGTASGADVKRSDSESTEATSN
ERPFNPVSAANLERLLMSSTGPQTPINGELKSNDSQDTAFQSSRNMPSDTSVASPDYSHS
QSSSPIANYQESGNSEEPHKAEEQQQLSIYQLDNPGSGNYVWETVISPDKFETSTFAKCE
RNDIAIINRELDAQDNQLIHTNEDFIAYAVHREPIIRVIEISTGKSFLLHNNSPNKFVSV
AWGNDSVIKNRLMAIDTTGQVLIFAVDIATSTSEIIFQLSGAQSLSDPIKSRFHWYPKSS
TRFAVALSKHIIFFDLDLLNNISFPIPRSINAIQQLPCFLIDTGISAKEYDFSYDGTVFA
TVDKDALIKIYTVPTTFPSTPDKRPVPSEVSPIAIFTTRMERGPSKNYEKPINLRFISTP
GTNNSRYLVIVYVMNQLITLFDLYSKRNIQTFRFNNRPTAATTTSFSQFSVDNERSTLLV
GNPPSNSIYFFLFAKDETVSEQAPIYNSTYELILASLNTSEPVPADAKFSVIVAKKFEKA
ACISFTACKILESEDKYCIVVSNTDGYEYYSIPTSILDKTGKTVRSLESVQNYDADIGGT
IDLTERHSTASPSTVNSGFSTPRSQATGFSKKKKDKGERFETKDKSSSVLSPSSYSASTF
DAIPMDSIVSNILASLEKSVHKNYESLRSQLLEYKAANEKHTEAILSVVSSTLTENTGKI
LESVVEKSMQVALKEEIANSVRNALKNNLEKIESFLENSIAELQNSVREDFDKQTSSLAQ
LRYSIQNVAHAQKESEVKYNELNEQVKTLEGYVETVLEKFNDLKIENKVPETAPDVVPSS
YPPAAESNVSVSSDTSTKDVEKQEPSSAEQPAQGIAESLRRLKEYVKAGSVKECVAEWCN
MPSVAGFDVLSEISYDRMLENCSNLLLLTFIYHISLLDSVDDDRLSKRMEYISRICLNID
VNDPKVETVVHPVLTLTREALLRQSEFFSPIFKRRLVVLLRALDGKISEISVASSN

>sp|Q99996|AKAP9_HUMAN A-kinase anchor protein 9 OS=Homo sapiens OX=9606 GN=AKAP9 PE=1 SV=4

MEDEERQKKLEAGKAKLAQFRQRKAQSDGQSPSKKQKKKRKTSSSKHDVSAHHDLNIDQS
QCNEMYINSSQRVESTVIPESTIMRTLHSGEITSHEQGFSVELESEISTTADDCSSEVNG
CSFVMRTGKPTNLLREEEFGVDDSYSEQGAQDSPTHLEMMESELAGKQHEIEELNRELEE
MRVTYGTEGLQQLQEFEAAIKQRDGIITQLTANLQQARREKDETMREFLELTEQSQKLQI
QFQQLQASETLRNSTHSSTAADLLQAKQQILTHQQQLEEQDHLLEDYQKKKEDFTMQISF
LQEKIKVYEMEQDKKVENSNKEEIQEKETIIEELNTKIIEEEKKTLELKDKLTTADKLLG
ELQEQIVQKNQEIKNMKLELTNSKQKERQSSEEIKQLMGTVEELQKRNHKDSQFETDIVQ
RMEQETQRKLEQLRAELDEMYGQQIVQMKQELIRQHMAQMEEMKTRHKGEMENALRSYSN
ITVNEDQIKLMNVAINELNIKLQDTNSQKEKLKEELGLILEEKCALQRQLEDLVEELSFS
REQIQRARQTIAEQESKLNEAHKSLSTVEDLKAEIVSASESRKELELKHEAEVTNYKIKL
EMLEKEKNAVLDRMAESQEAELERLRTQLLFSHEEELSKLKEDLEIEHRINIEKLKDNLG
IHYKQQIDGLQNEMSQKIETMQFEKDNLITKQNQLILEISKLKDLQQSLVNSKSEEMTLQ
INELQKEIEILRQEEKEKGTLEQEVQELQLKTELLEKQMKEKENDLQEKFAQLEAENSIL
KDEKKTLEDMLKIHTPVSQEERLIFLDSIKSKSKDSVWEKEIEILIEENEDLKQQCIQLN

EEIEKQRNTFSFAEKNFEVNYQELQEEYACLLKVKDDLEDSKNKQELEYKSKLKALNEEL
HLQRINPTTVKMKSSVFDEDKTFVAETLEMGEVVEKDTTELMEKLEVTKREKLELSQRLS
DLSEQLKQKHGEISFLNEEVKSLKQEKEQVSLRCRELEIIINHNRAENVQSCDTQVSSLL
DGVVTMTSRGAEGSVSKVNKSFGEESKIMVEDKVSFENMTVGEESKQEQLILDHLPSVTK
ESSLRATQPSENDKLQKELNVLKSEQNDLRLQMEAQRICLSLVYSTHVDQVREYMENEKD
KALCSLKEELIFAQEEKIKELQKIHQLELQTMKTQETGDEGKPLHLLIGKLQKAVSEECS
YFLQTLCSVLGEYYTPALKCEVNAEDKENSGDYISENEDPELQDYRYEVQDFQENMHTLL
NKVTEEYNKLLVLQTRLSKIWGQQTDGMKLEFGEENLPKEETEFLSIHSQMTNLEDIDVN
HKSKLSSLQDLEKTKLEEQVQELESLISSLQQQLKETEQNYEAEIHCLQKRLQAVSESTV
PPSLPVDSVVITESDAQRTMYPGSCVKKNIDGTIEFSGEFGVKEETNIVKLLEKQYQEQL
EEEVAKVIVSMSIAFAQQTELSRISGGKENTASSKQAHAVCQQEQHYFNEMKLSQDQIGF
QTFETVDVKFKEEFKPLSKELGEHGKEILLSNSDPHDIPESKDCVLTISEEMFSKDKTFI
VRQSIHDEISVSSMDASRQLMLNEEQLEDMRQELVRQYQEHQQATELLRQAHMRQMERQR
EDQEQLQEEIKRLNRQLAQRSSIDNENLVSERERVLLEELEALKQLSLAGREKLCCELRN
SSTQTQNGNENQGEVEEQTFKEKELDRKPEDVPPEILSNERYALQKANNRLLKILLEVVK
TTAAVEETIGRHVLGILDRSSKSQSSASLIWRSEAEASVKSCVHEEHTRVTDESIPSYSG
SDMPRNDINMWSKVTEEGTELSQRLVRSGFAGTEIDPENEELMLNISSRLQAAVEKLLEA
ISETSSQLEHAKVTQTELMRESFRQKQEATESLKCQEELRERLHEESRAREQLAVELSKA
EGVIDGYADEKTLFERQIQEKTDIIDRLEQELLCASNRLQELEAEQQQIQEERELLSRQK
EAMKAEAGPVEQQLLQETEKLMKEKLEVQCQAEKVRDDLQKQVKALEIDVEEQVSRFIEL
EQEKNTELMDLRQQNQALEKQLEKMRKFLDEQAIDREHERDVFQQEIQKLEQQLKVVPRF
QPISEHQTREVEQLANHLKEKTDKCSELLLSKEQLQRDIQERNEEIEKLEFRVRELEQAL
LVSADTFQKVEDRKHFGAVEAKPELSLEVQLQAERDAIDRKEKEITNLEEQLEQFREELE
NKNEEVQQLHMQLEIQKKESTTRLQELEQENKLFKDDMEKLGLAIKESDAMSTQDQHVLF
GKFAQIIQEKEVEIDQLNEQVTKLQQQLKITTDNKVIEEKNELIRDLETQIECLMSDQEC
VKRNREEEIEQLNEVIEKLQQELANIGQKTSMNAHSLSEEADSLKHQLDVVIAEKLALEQ
QVETANEEMTFMKNVLKETNFKMNQLTQELFSLKRERESVEKIQSIPENSVNVAIDHLSK
DKPELEVVLTEDALKSLENQTYFKSFEENGKGSIINLETRLLQLESTVSAKDLELTQCYK
QIKDMQEQGQFETEMLQKKIVNLQKIVEEKVAAALVSQIQLEAVQEYAKFCQDNQTISSE
PERTNIQNLNQLREDELGSDISALTLRISELESQVVEMHTSLILEKEQVEIAEKNVLEKE
KKLLELQKLLEGNEKKQREKEKKRSPQDVEVLKTTTELFHSNEESGFFNELEALRAESVA
TKAELASYKEKAEKLQEELLVKETNMTSLQKDLSQVRDHLAEAKEKLSILEKEDETEVQE
SKKACMFEPLPIKLSKSIASQTDGTLKISSSNQTPQILVKNAGIQINLQSECSSEEVTEI
ISQFTEKIEKMQELHAAEILDMESRHISETETLKREHYVAVQLLKEECGTLKAVIQCLRS
KEGSSIPELAHSDAYQTREICSSDSGSDWGQGIYLTHSQGFDIASEGRGEESESATDSFP
KKIKGLLRAVHNEGMQVLSLTESPYSDGEDHSIQQVSEPWLEERKAYINTISSLKDLITK
MQLQREAEVYDSSQSHESFSDWRGELLLALQQVFLEERSVLLAAFRTELTALGTTDAVGL
LNCLEQRIQEQGVEYQAAMECLQKADRRSLLSEIQALHAQMNGRKITLKREQESEKPSQE
LLEYNIQQKQSQMLEMQVELSSMKDRATELQEQLSSEKMVVAELKSELAQTKLELETTLK
AQHKHLKELEAFRLEVKDKTDEVHLLNDTLASEQKKSRELQWALEKEKAKLGRSEERDKE
ELEDLKFSLESQKQRNLQLNLLLEQQKQLLNESQQKIESQRMLYDAQLSEEQGRNLELQV
LLESEKVRIREMSSTLDRERELHAQLQSSDGTGQSRPPLPSEDLLKELQKQLEEKHSRIV
ELLNETEKYKLDSLQTRQQMEKDRQVHRKTLQTEQEANTEGQKKMHELQSKVEDLQRQLE
EKRQQVYKLDLEGQRLQGIMQEFQKQELEREEKRESRRILYQNLNEPTTWSLTSDRTRNW
VLQQKIEGETKESNYAKLIEMNGGGTGCNHELEMIRQKLQCVASKLQVLPQKASERLQFE
TADDEDFIWVQENIDEIILQLQKLTGQQGEEPSLVSPSTSCGSLTERLLRQNAELTGHIS
QLTEEKNDLRNMVMKLEEQIRWYRQTGAGRDNSSRFSLNGGANIEAIIASEKEVWNREKL
TLQKSLKRAEAEVYKLKAELRNDSLLQTLSPDSEHVTLKRIYGKYLRAESFRKALIYQKK
YLLLLLGGFQECEDATLALLARMGGQPAFTDLEVITNRPKGFTRFRSAVRVSIAISRMKF

LVRRWHRVTGSVSININRDGFGLNQGAEKTDSFYHSSGGLELYGEPRHTTYRSRSDLDYI
RSPLPFQNRYPGTPADFNPGSLACSQLQNYDPDRALTDYITRLEALQRRLGTIQSGSTTQ
FHAGMRR

>tr|H2L067|H2L067_CAEEL Muscle EXcess OS=Caenorhabditis elegans OX=6239 GN=mex-3 PE=1 SV=1

MPVVSVRPFSMRNEGFSNGHNTWNDAEFWKPMKEEQIAYKLPGAWYYEEDTASCSPVSDP
EDIAQFLNYRTSIGVQNVTESVEVPTSEHVAEIVGRQGCKIKALRAKTNTYIKTPVRGED
PIFVVTGRLEDVNEAKREIDCAAEHFTQIRASRRHTQGAHAPGQITSYVRVPLRVVGLVV
GPKGATIKRIQQDTHTYIITPSREREPVFEVTGLPHNVEAARKEIETHIFQRTGNLPETD
NDFAGQLAGVSLMVQKQQQAQQQMQEAQQQSMFYRRAFGNSNPFNQKEMSSSPFGMESSL
GLDALLRSFPSMRSSLTPESLSGTGLSSRPSLGGGQSAKQDLPTYDYWGTNNSLNDIMEN
EILSRKYDALSAWSSMGLEKREESPTNGLMSSLKGTSAGFGLLSTIWSGGNMNLSPGSLA
SASASPTSSTCDHNDHTLVPING

>sp|P38760|MIP6_YEAST RNA-binding protein MIP6 OS=Saccharomyces cerevisiae (strain ATCC 204508 / S288c) OX=559292 GN=MIP6 PE=1 SV=1

MPNSHGNVLNNISLNSKQNPRSISKSCPNDKDARQKSFKTISAQALVRVQGAGYKLGDVK
LKDAEVKEKNSLKKYDCKNATQEKKEQEQVFEKTVAKGSVQKYITKTSKTNSLFIGNLKS
TVTEEMLRKIFKRYQSFESAKVCRDFLTKKSLGYGYLNFKDKNDAESARKEFNYTVFFGQ
EVKIMPSMKNTLFRKNIGTNVFFSNLPLENPQLTTRSFYLIMIEYGNVLSCLLERRKNIG
FVYFDNDISARNVIKKYNNQEFFGNKIICGLHFDKEVRTRPEFTKRKKMIGSDIVIEDEL
LASNNLSDNARSKTILVKNLPSDTTQEEVLDYFSTIGPIKSVFISEKQANTPHKAFVTYK
NEEESKKAQKCLNKTIFKNHTIWVGPGKDKPVHNQIGTNKKTKVYLKNLSFNCNKEFISQ
LCLQEKIRFSEIKITNYNSLNWTFCGHVECFSRSDAERLFNILDRRLIGSSLVEASWSKN
NDNILNEIDYDDGNNNENYKKLINISSMMRFRTQELSAHQKGLTSQFQQVVSPFSSYSNS
YTNMNSLVATPMKPHPAFNLITNTVDEKLHQPKRTKQENAEILESLKKIINRNLQRISIS
GLNKEENLRSISEFIFDVFWEHDSERLSHFLLMTNTSLESQKILQKQVTRAAESLGFTV

>sp|G5EC37|SEPA1_CAEEL Protein sepa-1 OS=Caenorhabditis elegans OX=6239 GN=sepa-1 PE=1 SV=1

MTPLSALTSNPAPSPPPKFALGKCPATAIHVVSVLRPNRQRFCYEKTDAGDLIPHKCLIC
QPILTEKHISPYYAVYSDLLEDFVLFGYNTMTGKMEQFIYAFKTDCFVEVNRPEIKYNPA
FLVKGNVVVALNGPEGELVVIERDCRGLLSKESTSYGQFRTLPTAALRTLTLQDLERDRW

DRAANSDEVKISSGNESFDRLYAEYQKNLPRFQVRQCLHLNKEFLCVYSKTSGDYTRLEY
IDETGDFQKISCTLCTCEVTESNLIPLYVERNASELVIHVHNTENNQIEQYIYDVRTFGF
VQVKRNLVYDPKKITSGLNLFMAENIDNRKVYMIMRGRDGRLQKETSGSGGFEKMQPVAV
KTFQVQWVEMKTEFEKKKASTERVEPQHPVQTEGEDIMETVLAMVESFNCDLRKELGLTQ
DQEIPRKAPRVESAETEENIVKNLEKLQIAKDPEEPTTAASEGGNTYGYQELDDTMSEGL
LEKEAESKHQDANEPEPVKNVTYEPDVAAMDKKKKRRELKSRLNKINAQIDELEKRRMER
AGKKQVVSSSVPSEEAAQVEAPASPALAENTNQISNEETPKIDIFEGYNGSFLFGTNTSK
EWIVEDIRNHMVGKLLKAFWPRIQNVEEMNGELFKKLIANARKCETEILEASNDRDEYYR
LMQLTVDQILKKTLKKDQRATEHNHQQPTQSSDELAKNHEKN

>sp|Q15020|SART3_HUMAN Squamous cell carcinoma antigen recognized by T-cells 3 OS=Homo sapiens OX=9606 GN=SART3 PE=1 SV=1

MATAAETSASEPEAESKAGPKADGEEDEVKAARTRRKVLSRAVAAATYKTMGPAWDQQEE
GVSESDGDEYAMASSAESSPGEYEWEYDEEEEKNQLEIERLEEQLSINVYDYNCHVDLIR
LLRLEGELTKVRMARQKMSEIFPLTEELWLEWLHDEISMAQDGLDREHVYDLFEKAVKDY
ICPNIWLEYGQYSVGGIGQKGGLEKVRSVFERALSSVGLHMTKGLALWEAYREFESAIVE
AARLEKVHSLFRRQLAIPLYDMEATFAEYEEWSEDPIPESVIQNYNKALQQLEKYKPYEE
ALLQAEAPRLAEYQAYIDFEMKIGDPARIQLIFERALVENCLVPDLWIRYSQYLDRQLKV
KDLVLSVHNRAIRNCPWTVALWSRYLLAMERHGVDHQVISVTFEKALNAGFIQATDYVEI
WQAYLDYLRRRVDFKQDSSKELEELRAAFTRALEYLKQEVEERFNESGDPSCVIMQNWAR
IEARLCNNMQKARELWDSIMTRGNAKYANMWLEYYNLERAHGDTQHCRKALHRAVQCTSD
YPEHVCEVLLTMERTEGSLEDWDIAVQKTETRLARVNEQRMKAAEKEAALVQQEEEKAEQ
RKRARAEKKALKKKKKIRGPEKRGADEDDEKEWGDDEEEQPSKRRRVENSIPAAGETQNV
EVAAGPAGKCAAVDVEPPSKQKEKAASLKRDMPKVLHDSSKDSITVFVSNLPYSMQEPDT
KLRPLFEACGEVVQIRPIFSNRGDFRGYCYVEFKEEKSALQALEMDRKSVEGRPMFVSPC
VDKSKNPDFKVFRYSTSLEKHKLFISGLPFSCTKEELEEICKAHGTVKDLRLVTNRAGKP
KGLAYVEYENESQASQAVMKMDGMTIKENIIKVAISNPPQRKVPEKPETRKAPGGPMLLP
QTYGARGKGRTQLSLLPRALQRPSAAAPQAENGPAAAPAVAAPAATEAPKMSNADFAKLF
LRK

>sp|P07222|NPM_XENLA Nucleophosmin OS=Xenopus laevis OX=8355 GN=npm1 PE=1 SV=1

MEDSMDMDNIAPLRPQNFLFGCELKADKKEYSFKVEDDENEHQLSLRTVSLGASAKDELH
VVEAEGINYEGKTIKIALASLKPSVQPTVSLGGFEITPPVILRLKSGSGPVYVSGQHLVA
LEDLESSDDEDEEHEPSPKNAKRIAPDSASKVPRKKTRLEEEEEDSDEDDDDDEDDDDED
DDEEEEETPVKKTDSTKSKAAQKLNHNGKASALSTTQKTPKTPEQKGKQDTKPQTPKTPK
TPLSSEEIKAKMQTYLEKGNVLPKVEVKFANYVKNCFRTENQKVIEDLWKWRQSLKDGK

>sp|P29227|HP1_DROVI Heterochromatin protein 1 OS=Drosophila virilis OX=7244 GN=HP1A PE=3 SV=1

MGKKTDNPETNNASSGAEEEEEEYAVEKILDRRVRKGKVEYYLKWKGYAETENTWEPEGN
LDCQDLIQQYELSRKDEANAAASSSSSSSKKERPGSSTKVKETGRTSTTASNSSGSKRKS
EEPAGPAGSKSKRVESEDTGDIVPAGGTGFDRGLEAEKILGASDNNGRLTFLIQFKGVDQ
AEMVPSTVANVKIPQMVIRFYEERLSWYSDNED

>sp|Q9NRC8|SIR7_HUMAN NAD-dependent protein deacetylase sirtuin-7 OS=Homo sapiens OX=9606 GN=SIRT7 PE=1 SV=1

MAAGGLSRSERKAAERVRRLREEQQRERLRQVSRILRKAAAERSAEEGRLLAESADLVTE
LQGRSRRREGLKRRQEEVCDDPEELRGKVRELASAVRNAKYLVVYTGAGISTAASIPDYR
GPNGVWTLLQKGRSVSAADLSEAEPTLTHMSITRLHEQKLVQHVVSQNCDGLHLRSGLPR
TAISELHGNMYIEVCTSCVPNREYVRVFDVTERTALHRHQTGRTCHKCGTQLRDTIVHFG
ERGTLGQPLNWEAATEAASRADTILCLGSSLKVLKKYPRLWCMTKPPSRRPKLYIVNLQW
TPKDDWAALKLHGKCDDVMRLLMAELGLEIPAYSRWQDPIFSLATPLRAGEEGSHSRKSL
CRSREEAPPGDRGAPLSSAPILGGWFGRGCTKRTKRKKVT

>sp|Q16533|SNPC1_HUMAN snRNA-activating protein complex subunit 1 OS=Homo sapiens OX=9606 GN=SNAPC1 PE=1 SV=1

MGTPPGLQTDCEALLSRFQETDSVRFEDFTELWRNMKFGTIFCGRMRNLEKNMFTKEALA
LAWRYFLPPYTFQIRVGALYLLYGLYNTQLCQPKQKIRVALKDWDEVLKFQQDLVNAQHF
DAAYIFRKLRLDRAFHFTAMPKLLSYRMKKKIHRAEVTEEFKDPSDRVMKLITSDVLEEM
LNVHDHYQNMKHVISVDKSKPDKALSLIKDDFFDNIKNIVLEHQQWHKDRKNPSLKSKTN
DGEEKMEGNSQETERCERAESLAKIKSKAFSVVIQASKSRRHRQVKLDSSDSDSASGQGQ
VKATRKKEKKERLKPAGRKMSLRNKGNVQNIHKEDKPLSLSMPVITEEEENESLSGTEFT
ASKKRRKH

Sequences from the LLPS positive set which are classified correctly by PScore and incorrectly by score s_4, against the negative set hsnLLPS, with a false positive FPR=0.3 as the precision threshold (set$\tilde{\mathcal{S}}_P$)

The sequence stretches selected by PASTA for the best β-pairing are highlighted in yellow background. All pairings are predicted to be in-register parallel, except for the 3 sequences marked by ***, for which in register anti-parallel pairing is predicted. Sequences are ranked from the highest to the lowest value of PScore.

>tr|A0A0G2UMW8|A0A0G2UMW8_DOSGI Histidine rich beak protein 1 OS=Dosidicus gigas OX=346249 GN=HBP-1 PE=2 SV=1

MKCVSFAIFLVVAIFGANSQLYGAPAVGGVVENAVNAAESGAAATHDAQGAYAEADTAGV
LDVNHAEHHDGVHDASGYGFGGLAGHGGFAGHGLYGPGFAGHGLLGAGYAGLGLHGAGFA
GHGLHGAGFAGHGLYGAGFAGHGLHGFAGHGLYGAGFAGHGLGLGGLHGALGHGALAHY

>sp|P40070|LSM4_YEAST U6 snRNA-associated Sm-like protein LSm4 OS=Saccharomyces cerevisiae (strain ATCC 204508 / S288c) OX=559292 GN=LSM4 PE=1 SV=1

MLPLYLLTNAKGQQMQIELKNGEIIQGILTNVDNWMNLTLSNVTEYSEESAINSEDNAES
SKAVKLNEIYIRGTFIKFIKLQDNIIDKVKQQINSNNNSNSNGPGHKRYYNNRDSNNNRG
NYNRRNNNNGNSNRRPYSQNRQYNNSNSSNINNSINSINSNNQNMNNGLGGSVQHHFNSS
SPQKVEF

>sp|P25644|PAT1_YEAST DNA topoisomerase 2-associated protein PAT1 OS=Saccharomyces cerevisiae (strain ATCC 204508 / S288c) OX=559292 GN=PAT1 PE=1 SV=4

MSFFGLENSGNARDGPLDFEESYKGYGEHELEENDYLNDETFGDNVQVGTDFDFGNPHSS
GSSGNAIGGNGVGATARSYVAATAEGISGPRTDGTAAAGPLDLKPMESLWSTAPPPAMAP
SPQSTMAPAPAPQQMAPLQPILSMQDLERQQRQMQQQFMNFHAMGHPQGLPQGPPQQQFP
MQPASGQPGPSQFAPPPPPPGVNVNMNQMPMGPVQVPVQASPSPIGMSNTPSPGPVVGAT
KMPLQSGRRSKRDLSPEEQRRLQIRHAKVEKILKYSGLMTPRDKDFITRYQLSQIVTEDP
YNEDFYFQVYKIIQRGGITSESNKGLIARAYLEHSGHRLGGRYKRTDIALQRMQSQVEKA
VTVAKERPSKLKDQQAAAGNSSQDNKQANTVLGKISSTLNSKNPRRQLQIPRQQPSDPDA

LKDVTDSLTNVDLASSGSSSTGSSAAAVASKQRRRSSYAFNNGNGATNLNKSGGKKFILE
LIETVYEEILDLEANLRNGQQTDSTAMWEALHIDDSSYDVNPFISMLSFDKGIKIMPRIF
NFLDKQQKLKILQKIFNELSHLQIIILSSYKTTPKPTLTQLKKVDLFQMIILKIIVSFLS
NNSNFIEIMGLLLQLIRNNNVSFLTTSKIGLNLITILISRAALIKQDSSRSNILSSPEIS
TWNEIYDKLFTSLESKIQLIFPPREYNDHIMRLQNDKFMDEAYIWQFLASLALSGKLNHQ
RIIIDEVRDEIFATINEAETLQKKEKELSVLPQRSQELDTELKSIIYNKEKLYQDLNLFL
NVMGLVYRDGEISELK

>sp|P05067|A4_HUMAN Amyloid-beta precursor protein OS=Homo sapiens OX=9606 GN=APP PE=1 SV=3

MLPGLALLLLAAWTARALEVPTDGNAGLLAEPQIAMFCGRLNMHMNVQNGKWDSDPSGTK
TCIDTKEGILQYCQEVYPELQITNVVEANQPVTIQNWCKRGRKQCKTHPHFVIPYRCLVG
EFVSDALLVPDKCKFLHQERMDVCETHLHWHTVAKETCSEKSTNLHDYGMLLPCGIDKFR
GVEFVCCPLAEESDNVDSADAEEDDSDVWWGGADTDYADGSEDKVVEVAEEEEVAEVEEE
EADDDEDDEDGDEVEEEAEEPYEEATERTTSIATTTTTTTESVEEVVREVCSEQAETGPC
RAMISRWYFDVTEGKCAPFFYGGCGGNRNNFDTEEYCMAVCGSAMSQSLLKTTQEPLARD
PVKLPTTAASTPDAVDKYLETPGDENEHAHFQKAKERLEAKHRERMSQVMREWEEAERQA
KNLPKADKKAVIQHFQEKVESLEQEAANERQQLVETHMARVEAMLNDRRRLALENYITAL
QAVPPRPRHVFNMLKKYVRAEQKDRQHTLKHFEHVRMVDPKKAAQIRSQVMTHLRVIYER
MNQSLSLLYNVPAVAEEIQDEVDELLQKEQNYSDDVLANMISEPRISYGNDALMPSLTET
KTTVELLPVNGEFSLDDLQPWHSFGADSVPANTENEVEPVDARPAADRGLTTRPGSGLTN
IKTEEISEVKMDAEFRHDSGYEVHHQKLVFFAEDVGSNKGAIIGLMVGGVVIATVIVITL
VMLKKKQYTSIHHGVVEVDAAVTPEERHLSKMQQNGYENPTYKFFEQMQN

>sp|O43561|LAT_HUMAN Linker for activation of T-cells family member 1 OS=Homo sapiens OX=9606 GN=LAT PE=1 SV=1

MEEAILVPCVLGLLLLPILAMLMALCVHCHRLPGSYDSTSSDSLYPRGIQFKRPHTVAPW
PPAYPPVTSYPPLSQPDLLPIPRSPQPLGGSHRTPSSRRDSDGANSVASYENEGASGIRG
AQAGWGVWGPSWTRLTPVSLPPEPACEDADEDEDDYHNPGYLVVLPDSTPATSTAAPSAP
ALSTPGIRDSAFSMESIDDYVNVPESGESAEASLDGSREYVNVSQELHPGAAKTEPAALS
SQEAEEVEEEGAPDYENLQELN

>sp|P18573|GDA9_WHEAT Alpha/beta-gliadin MM1 OS=Triticum aestivum OX=4565 PE=1 SV=1

MKTFLILALLAIVATTARIAVRVPVPQLQPQNPSQQQPQEQVPLVQQQQFPGQQQPFPPQ

QPYPQPQPFPSQQPYLQLQPFPQPQLPYPQPQLPYPQPQLPYPQPQPFRPQQPYPQSQPQ
YSQPQQPISQQQQQQQQQQQQKQQQQQQQQILQQILQQQLIPCRDVVLQQHSIAYGSSQV
LQQSTYQLVQQLCCQQLWQIPEQSRCQAIHNVVHAIILHQQQQQQQQQQQQPLSQVSFQQ
PQQQYPSGQGSFQPSQQNPQAQGSVQPQQLPQFEEIRNLALETLPAMCNVYIPPYCTIAP
VGIFGTN

>sp|P52298|NCBP2_HUMAN Nuclear cap-binding protein subunit 2 OS=Homo sapiens OX=9606 GN=NCBP2 PE=1 SV=1

MSGGLLKALRSDSYVELSQYRDQHFRGDNEEQEKLLKKSCTLYVGNLSFYTTEEQIYELF
SKSGDIKKIIMGLDKMKKTACGFCFVEYYSRADAENAMRYINGTRLDDRIIRTDWDAGFK
EGRQYGRGRSGGQVRDEYRQDYDAGRGGYGKLAQNQ

>sp|Q9H4Z2|ZN335_HUMAN Zinc finger protein 335 OS=Homo sapiens OX=9606 GN=ZNF335 PE=1 SV=1

MEENEVESSSDAAPGPGRPEEPSESGLGVGTSEAVSADSSDAAAAPGQAEADDSGVGQSS
DRGSRSQEEVSESSSSADPLPNSYLPDSSSVSHGPVAGVTGGPPALVHSSALPDPNMLVS
DCTASSSDLGSAIDKIIESTIGPDLIQNCITVTSAEDGGAETTRYLILQGPDDGAPMTSP
MSSSTLAHSLAAIEALADGPTSTSTCLEAQGGPSSPVQLPPASGAEEPDLQSLEAMMEVV
VVQQFKCKMCQYRSSTKATLLRHMRERHFRPVAAAAAAAGKKGRLRKWSTSTKSQEEEGP
EEEDDDDIVDAGAIDDLEEDSDYNPAEDEPRGRQLRLQRPTPSTPRPRRRPGRPRKLPRL
EISDLPDGVEGEPLVSSQSGQSPPEPQDPEAPSSSGPGHLVAMGKVSRTPVEAGVSQSDA
ENAAPSCPDEHDTLPRRRGRPSRRFLGKKYRKYYYKSPKPLLRPFLCRICGSRFLSHEDL
RFHVNSHEAGDPQLFKCLQCSYRSRRWSSLKEHMFNHVGSKPYKCDECSYTSVYRKDVIR
HAAVHSRDRKKRPDPTPKLSSFPCPVCGRVYPMQKRLTQHMKTHSTEKPHMCDKCGKSFK
KRYTFKMHLLTHIQAVANRRFKCEFCEFVCEDKKALLNHQLSHVSDKPFKCSFCPYRTFR
EDFLLSHVAVKHTGAKPFACEYCHFSTRHKKNLRLHVRCRHASSFEEWGRRHPEEPPSRR
RPFFSLQQIEELKQQHSAAPGPPPSSPGPPEIPPEATTFQSSEAPSLLCSDTLGGATIIY
QQGAEESTAMATQTALDLLLNMSAQRELGGTALQVAVVKSEDVEAGLASPGGQPSPEGAT
PQVVTLHVAEPGGGAAAESQLGPPDLPQITLAPGPFGGTGYSVITAPPMEEGTSAPGTPY
SEEPAGEAAQAVVVSDTLKEAGTHYIMATDGTQLHHIELTADGSISFPSPDALASGAKWP
LLQCGGLPRDGPEPPSPAKTHCVGDSQSSASSPPATSKALGLAVPPSPPSAATAASKKFS
CKICAEAFPGRAEMESHKRAHAGPGAFKCPDCPFSARQWPEVRAHMAQHSSLRPHQCSQC
SFASKNKKDLRRHMLTHTKEKPFACHLCGQRFNRNGHLKFHIQRLHSPDGRKSGTPTARA
PTQTPTQTIILNSDDETLATLHTALQSSHGVLGPERLQQALSQEHIIVAQEQTVTNQEEA
AYIQEITTADGQTVQHLVTSDNQVQYIISQDGVQHLLPQEYVVVPEGHHIQVQEGQITHI
QYEQGAPFLQESQIQYVPVSPGQQLVTQAQLEAAAHSAVTAVADAAMAQAQGLFGTDETV
PEHIQQLQHQGIEYDVITLADD

>sp|Q9UER7|DAXX_HUMAN Death domain-associated protein 6 OS=Homo sapiens OX=9606 GN=DAXX PE=1 SV=2

MATANSIIVLDDDDEDEAAAQPGPSHPLPNAASPGAEAPSSSEPHGARGSSSSGGKKCYK
LENEKLFEEFLELCKMQTADHPEVVPFLYNRQQRAHSLFLASAEFCNILSRVLSRARSRP
AKLYVYINELCTVLKAHSAKKKLNLAPAATTSNEPSGNNPPTHLSLDPTNAENTASQSPR
TRGSRRQIQRLEQLLALYVAEIRRLQEKELDLSELDDPDSAYLQEARLKRKLIRLFGRLC
ELKDCSSLTGRVIEQRIPYRGTRYPEVNRRIERLINKPGPDTFPDYGDVLRAVEKAAARH
SLGLPRQQLQLMAQDAFRDVGIRLQERRHLDLIYNFGCHLTDDYRPGVDPALSDPVLARR
LRENRSLAMSRLDEVISKYAMLQDKSEEGERKKRRARLQGTSSHSADTPEASLDSGEGPS
GMASQGCPSASRAETDDEDDEESDEEEEEEEEEEEEEATDSEEEEDLEQMQEGQEDDEEE
DEEEEAAAGKDGDKSPMSSLQISNEKNLEPGKQISRSSGEQQNKGRIVSPSLLSEEPLAP
SSIDAESNGEQPEELTLEEESPVSQLFELEIEALPLDTPSSVETDISSSRKQSEEPFTTV
LENGAGMVSSTSFNGGVSPHNWGDSGPPCKKSRKEKKQTGSGPLGNSYVERQRSVHEKNG
KKICTLPSPPSPLASLAPVADSSTRVDSPSHGLVTSSLCIPSPARLSQTPHSQPPRPGTC
KTSVATQCDPEEIIVLSDSD

>tr|A8IGD9|A8IGD9_CHLRE Low-CO2-inducible protein OS=Chlamydomonas reinhardtii OX=3055 GN=LCI5 PE=4 SV=1

MATISSMRVGAASRVVVSGRVKTVKVAARGSWRESSTATVQASRASSATNRVSPTRSVLP
ANWRQELESLRNGNGSSSAASSAPAPARSSSASWRDAAPASSAPARSSSASKKAVTPSRS
ALPSNWKQELESLRSSSPAPASSAPAPARSSSASWRDAAPASSAPARSSSSKKAVTPSLS
APPCPLTRSWRACAAAPPPPPRRPPPRPAPRRPAGVTPPRPRRPLPAPALPPRRP

>sp|Q9BJZ5|FUSIL_DROME RNA-binding protein fusilli OS=Drosophila melanogaster OX=7227 GN=fus PE=2 SV=1

MQVPEHVVSLYIATCGQNGSGLGSDEKEIILLVFVLLEVSTGQIVGTKQILVRPDGYFIK
DRTISSSSDNSSVTNNTASSPPLAIGDANNGSGSTSGNGNSLENGSELILPIAEAQAAGK
PLSEAIEEFDAYLRSLSLHDTEIKLVTDGQLPLRQCLHREASAKDVELPAYYNRFSDLRK
EFLRYKSGDLARALVPVKDVKKMLQAPTLPMPQSIAEMLGELNISSVEDNDFYIRESRDM
VTVIQTLLQAGHKFAANELVNLVLEPGICSIDDEVDGNCIVRARGLPWQSSDQDIAKFFR
GLNVAKGGVALCLSPLGRRNGEALIRFVCQEHRDMALKRHKHHIGTRYIEVYRASGEDFL
AIAGGASNEAQAFLSKGAQVIIRMRGLPYDATAKQVLDFFTTGDTPPCHVLDGNEGVLFV
KKPDGRATGDAFVLFANETDAPKALGRHRESIGQRYIELFRSTTAEVQQVLNRSMDPKNY
ESGGGHSQPPLIAQLPTMQLPLLPQVGAHSLSHSLGASPANLCPPVPPPALPLPTQHLIT
SGTTKNCIRLRGLPYEAMVEHILHFLDDFAKHIIYQGVHMVINAQGQPSGEAFIQMDLEE

SARLCAQRRHNHYMMFGKKYRYIEVFQCSGDDMNMVLNGGLASPVAQPPPPHLGHAHKQQ
SLLAATTGMFSSAGQSPTTVAAGTAQSPLGGTHTHTHPHSHAHAHATGHAHAAAAAAHHG
LSASSAMLPGLSAAASASGLASFMSAGQQSAAAAAHSAASLQNAAVALAPQGYALNPFSL
PPPGSAAAAAAASTPALLAQQQAQFIAQQSLLVRQQAAAAALAAEQQQQQQQQQLYASAM
LQQHPLYLQQQQQQQQLYASAMLQQGQPQFVLMQRPSAAYLPPFPLSYMSAAGAASGVGV
APGAAVAGAAAAASPSASNSSLSQSMKRSYENAFQQEAAGAAAAASAAKRALTRQPSSVY
SYYNSGI

>sp|O65934|ABC1_MYCTU ABC transporter ATP-binding/permease protein Rv1747 OS=Mycobacterium tuberculosis (strain ATCC 25618 / H37Rv) OX=83332 GN=Rv1747 PE=1 SV=1

MPMSQPAAPPVLTVRYEGSERTFAAGHDVVVGRDLRADVRVAHPLISRAHLLLRFDQGRW
VAIDNGSLNGLYLNNRRVPVVDIYDAQRVHIGNPDGPALDFEVGRHRGSAGRPPQTTSIR
LPNLSAGAWPTDGPPQTGTLGSGQLQQLPPATTRIPAAPPSGPQPRYPTGGQQLWPPSGP
QRAPQIYRPPTAAPPPAGARGGTEAGNLATSMMKILRPGRLTGELPPGAVRIGRANDNDI
VIPEVLASRHHATLVPTPGGTEIRDNRSINGTFVNGARVDAALLHDGDVVTIGNIDLVFA
DGTLARREENLLETRVGGLDVRGVTWTIDGDKTLLDGISLTARPGMLTAVIGPSGAGKST
LARLVAGYTHPTDGTVTFEGHNVHAEYASLRSRIGMVPQDDVVHGQLTVKHALMYAAELR
LPPDTTKDDRTQVVARVLEELEMSKHIDTRVDKLSGGQRKRASVALELLTGPSLLILDEP
TSGLDPALDRQVMTMLRQLADAGRVVLVVTHSLTYLDVCDQVLLLAPGGKTAFCGPPTQI
GPVMGTTNWADIFSTVADDPDAAKARYLARTGPTPPPPPVEQPAELGDPAHTSLFRQFST
IARRQLRLIVSDRGYFVFLALLPFIMGALSMSVPGDVGFGFPNPMGDAPNEPGQILVLLN
VGAVFMGTALTIRDLIGERAIFRREQAVGLSTTAYLIAKVCVYTVLAVVQSAIVTVIVLV
GKGGPTQGAVALSKPDLELFVDVAVTCVASAMLGLALSAIAKSNEQIMPLLVVAVMSQLV
FSGGMIPVTGRVPLDQMSWVTPARWGFAASAATVDLIKLVPGPLTPKDSHWHHTASAWWF
DMAMLVALSVIYVGFVRWKIRLKAC

>sp|P55072|TERA_HUMAN Transitional endoplasmic reticulum ATPase OS=Homo sapiens OX=9606 GN=VCP PE=1 SV=4

MASGADSKGDDLSTAILKQKNRPNRLIVDEAINEDNSVVSLSQPKMDELQLFRGDTVLLK
GKKRREAVCIVLSDDTCSDEKIRMNRVVRNNLRVRLGDVISIQPCPDVKYGKRIHVLPID
DTVEGITGNLFEVYLKPYFLEAYRPIRKGDIFLVRGGMRAVEFKVVETDPSPYCIVAPDT
VIHCEGEPIKREDEEESLNEVGYDDIGGCRKQLAQIKEMVELPLRHPALFKAIGVKPPRG
ILLYGPPGTGKTLIARAVANETGAFFFLINGPEIMSKLAGESESNLRKAFEEAEKNAPAI
IFIDELDAIAPKREKTHGEVERRIVSQLLTLMDGLKQRAHVIVMAATNRPNSIDPALRRF
GRFDREVDIGIPDATGRLEILQIHTKNMKLADDVDLEQVANETHGHVGADLAALCSEAAL
QAIRKKMDLIDLEDETIDAEVMNSLAVTMDDFRWALSQSNPSALRETVVEVPQVTWEDIG
GLEDVKRELQELVQYPVEHPDKFLKFGMTPSKGVLFYGPPGCGKTLLAKAIANECQANFI
SIKGPELLTMWFGESEANVREIFDKARQAAPCVLFFDELDSIAKARGGNIGDGGGAADRV
INQILTEMDGMSTKKNVFIIGATNRPDIIDPAILRPGRLDQLIYIPLPDEKSRVAILKAN

LRKSPVAKDVDLEFLAKMTNGFSGADLTEICQRACKLAIRESIESEIRRERERQTNPSAM
EVEEDDPVPEIRRDHFEEAMRFARRSVSDNDIRKYEMFAQTLQQSRGFGSFRFPSGNQGG
AGPSQGSGGGTGGSVYTEDNDDDLYG

>sp|Q9D415|DLGP1_MOUSE Disks large-associated protein 1 OS=Mus musculus OX=10090 GN=Dlgap1 PE=1 SV=3 ***

MKGLSGSRSHHHGITCEAACDSLSHHSDHKPYLLSPVDHHPADHPYYTQRNSFQAECVGP
FSDPLASSTFPRRHYTSQQELKDESALVPRTLATKANRLPTNLLDQFERQLPLSRDGYHT
LQYKRTAVEHRSDSPGRIRHLVHSVQKLFTKSHSLEGPSKGSVNGGKASPDESQTLRYGK
RSKSKERRSESKARSNASNASPTSPSWWSSDDNLDGDMCLYHTPSGVMTMGRCPDRSASQ
YFMEAYNTISEQAVKASRSNNDIKCSTCANLPVTLDAPLLKKSAWSSTLTVSRAREVYQK
ASVNMDQAMVKSEACQQERSCQYLQVPQDEWSGYTPRGKDDEIPCRRMRSGSYIKAMGDE
DSGDSDTSPKPSPKVAARRESYLKATQPSLTELTTLKISNEHSPKLQIRSHSYLRAVSEV
SINRSLDSLDPAGLLTSPKFRSRNESYMRAMSTISQVSEMEVNGQFESVCESVFSELESQ
AVEALDLPLPGCFRMRSHSYVRAIEKGCSQDDECVSLRSSSPPRTTTTVRTIQSSTGVIK
LSSAVEVSSCITTYKKTPPPVPPRTTTKPFISITAQSSTESAQDAYMDGQGQRGDMISQS
GLSNSTESLDSMKALTAAIEAANAQIHGPASQHMGSNAAAVTTTTTIATVTTEDRKKDFK
KNRCLSIGIQVDDAEEPEKMAESKTSNKFQSVGVQVEEEKCFRRFTRSNSVTTAVQADLD
FHDNLENSLESIEDNSCPGPMARQFSRDASTSTVSIQGSGNHYHACAADDDFDTDFDPSI
LPPPDPWIDSITEDPLEAVQRSVCHRDGHWFLKLLQAERDRMEGWCKLMEREERENNLPE
DILGKIRTAVGSAQLLMAQKFYQFRELCEENLNPNAHPRPTSQDLAGFWDMLQLSIENIS
MKFDELHQLKANNWKQMDPLDKKERRAPPPVPKKPAKGPAPLIRERSLESSQRQEARKRL
MAAKRAASVRQNSATESAESIEIYIPEAQTRL

>sp|O43663|PRC1_HUMAN Protein regulator of cytokinesis 1 OS=Homo sapiens OX=9606 GN=PRC1 PE=1 SV=2

MRRSEVLAEESIVCLQKALNHLREIWELIGIPEDQRLQRTEVVKKHIKELLDMMIAEEES
LKERLIKSISVCQKELNTLCSELHVEPFQEEGETTILQLEKDLRTQVELMRKQKKERKQE
LKLLQEQDQELCEILCMPHYDIDSASVPSLEELNQFRQHVTTLRETKASRREEFVSIKRQ
IILCMEALDHTPDTSFERDVVCEDEDAFCLSLENIATLQKLLRQLEMQKSQNEAVCEGLR
TQIRELWDRLQIPEEEREAVATIMSGSKAKVRKALQLEVDRLEELKMQNMKKVIEAIRVE
LVQYWDQCFYSQEQRQAFAPFCAEDYTESLLQLHDAEIVRLKNYYEVHKELFEGVQKWEE
TWRLFLEFERKASDPNRFTNRGGNLLKEEKQRAKLQKMLPKLEEELKARIELWEQEHSKA
FMVNGQKFMEYVAEQWEMHRLEKERAKQERQLKNKKQTETEMLYGSAPRTPSKRRGLAPN
TPGKARKLNTTTMSNATANSSIRPIFGGTVYHSPVSRLPPSGSKPVAASTCSGKKTPRTG
RHGANKENLELNGSILSGGYPGSAPLQRNFSINSVASTYSEFAKDPSLSDSSTVGLQREL
SKASKSDATSGILNSTNIQS

>sp|Q14781|CBX2_HUMAN Chromobox protein homolog 2 OS=Homo sapiens OX=9606 GN=CBX2 PE=1 SV=2

MEELSSVGEQVFAAECILSKRLRKGKLEYLVKWRGWSSKHNSWEPEENILDPRLLLAFQK
KEHEKEVQNRKRGKRPRGRPRKLTAMSSCSRRSKLKEPDAPSKSKSSSSSSSSTSSSSSS
DEEDDSDLDAKRGPRGRETHPVPQKKAQILVAKPELKDPIRKKRGRKPLPPEQKATRRPV
SLAKVLKTARKDLGAPASKLPPPLSAPVAGLAALKAHAKEACGGPSAMATPENLASLMKG
MASSPGRGGISWQSSIVHYMNRMTQSQAQAASRLALKAQATNKCGLGLDLKVRTQKGELG
MSPPGSKIPKAPSGGAVEQKVGNTGGPPHTHGASRVPAGCPGPQPAPTQELSLQVLDLQS
VKNGMPGVGLLARHATATKGVPATNPAPGKGTGSGLIGASGATMPTDTSKSEKLASRAVA
PPTPASKRDCVKGSATPSGQESRTAPGEARKAATLPEMSAGEESSSDSDPDSASPPSTG
QNPSVSVQTSQDWKPTRSLIEHVFVTDVTANLITVTVKESPTSVGFFNLRHY

>sp|Q86SG3|DAZ4_HUMAN Deleted in azoospermia protein 4 OS=Homo sapiens OX=9606 GN=DAZ4 PE=1 SV=2

MSAANPETPNSTISREASTQSSSAAASQGWVLPEGKIVPNTVFVGGIDARMDETEIGSCF
GRYGSVKEVKIITNRTGVSKGYGFVSFVNDVDVQKIVGSQIHFHGKKLKLGPAIRKQKLC
ARHVQPRPLVVNPPPPPQFQNVWRNPNTETYLQPQITPNPVTQHVQSAANPETPNSTISR
EASTQSSSAAASQGWVLPEGKIVPNTVFVGGIDARMDETEIGSCFGRYGSVKEVKIITNR
TGVSKGYGFVSFVNDVDVQKIVGSQIHFHGKKLKLGPAIRKQKLCARHVQPRPLVVNPPP
PPQFQNVWRNPNTETYLQPQITPNPVTQHVQAYSAYPHSPGQVITGCQLLVYNYQEYPTY
PDSAFQVTTGYQLPVYNYQPFPAYPRSPFQVTAGYQLPVYNYQAFPAYPNSPFQVATGYQ
FPVYNYQPFPAYPSSPFQVTAGYQLPVYNYQAFPAYPNSPFQVATGYQFPVYNYQAFPAY
PNSPVQVTTGYQLPVYNYQAFPAYPSSPFQVTTGYQLPVYNYQAFPAYPNSAVQVTTGYQ
FHVYNYQMPPQCPVGEQRRNLWTEAYKWWYLVCLIQRRD

>sp|Q9NQZ3|DAZ1_HUMAN Deleted in azoospermia protein 1 OS=Homo sapiens OX=9606 GN=DAZ1 PE=1 SV=2

MSAANPETPNSTISREASTQSSSAAASQGWVLPEGKIVPNTVFVGGIDARMDETEIGSCF
GRYGSVKEVKIITNRTGVSKGYGFVSFVNDVDVQKIVGSQIHFHGKKLKLGPAIRKQKLC
ARHVQPRPLVVNPPPPPQFQNVWRNPNTETYLQPQITPNPVTQHVQSAANPETPNSTISR
EASTQSSSAAASQGWVLPEGKIVPNTVFVGGIDARMDETEIGSCFGRYGSVKEVKIITNR
TGVSKGYGFVSFVNDVDVQKIVGSQIHFHGKKLKLGPAIRKQKLCARHVQPRPLVVNPPP
PPQFQNVWRNPNTETYLQPQITPNPVTQHVQSAANPETPNSTISREASTQSSSAAASQGW
VLPEGKIVPNTVFVGGIDARMDETEIGSCFGRYGSVKEVKIITNRTGVSKGYGFVSFVND
VDVQKIVGSQIHFHGKKLKLGPAIRKQKLCARHVQPRPLVVNPPPPPQFQNVWRNPNTET

YLQPQITPNPVTQHVQAYSAYPHSPGQVITGCQLLVYNYQEYPTYPDSAFQVTTGYQLPV
YNYQPFPAYPRSPFQVTAGYQLPVYNYQAFPAYPNSPFQVATGYQFPVYNYQPFPAYPSS
PFQVTAGYQLPVYNYQAFPAYPNSPFQVATGYQFPVYNYQAFPAYPNSPVQVTTGYQLPV
YNYQAFPAYPSSPFQVTTGYQLPVYNYQAFPAYPNSAVQVTTGYQFHVYNYQMPPQCPVG
EQRRNLWTEAYKWWYLVCLIQRRD

>sp|P40337|VHL_HUMAN von Hippel-Lindau disease tumor suppressor OS=Homo sapiens OX=9606 GN=VHL PE=1 SV=2 ***

MPRRAENWDEAEVGAEEAGVEEYGPEEDGGEESGAEESGPEESGPEELGAEEEMEAGRPR
PVLRSVNSREPSQVIFCNRSPRVVLPVWLNFDGEPQPYPTLPPGTGRRIHSYRGHLWLFR
DAGTHDGLLVNQTELFVPSLNVDGQPIFANITLPVYTLKERCLQVVRSLVKPENYRRLDI
VRSLYEDLEDHPNVQKDLERLTQERIAHQRMGD

>sp|P78953|MID1_SCHPO Division mal foutue 1 protein OS=Schizosaccharomyces pombe (strain 972 / ATCC 24843) OX=284812 GN=mid1 PE=1 SV=1

MKEQEFSYREAKDVSLDSKGLENSFLSSPNREKTPLFFEGNSNETSGYDQTKNFTHGDGD
MSLGNLSELNVATDLLESLDLRSMYMHGYGHLDSSFSSQHSPDNRKRMSSTSVFKRINSE
EEGRIPSLTYSAGTMNSTSSSTASLKGADIVADYETFNPDQNLAELSFDRSKSSRKRAVE
VAEFSRAKTMSPLEYTVQHPYQSHNELSTNPARARAGSVPNLARIPSDVKPVPPAHLSAS
STVGPRILPSLPKDTTEDNPALERVETTASLDMDYKPLEPLAPIQEAPVEDTSEPFSSVP
EATLDDSDISTESLRKKVLAKMEAKRISSGSSYASTLRKVYDFSELSLPTNGKDYDELYL
QSSRNSEPEISTIINDSLQQENMDEDISATSIPKSQAAYGHGSVTYHEVPRYNLTSASVG
YSISSQRGRIKSSSTIDNLSAILSSEDLRHPSMQPVPGTKRTYSNYCENEPNKSSQSLVS
SESHNVEGWNYSETGTVGFYDPSAEISASIDELRQSTPVARDSELLSRAHSFDLNRLDLP
SQDKSTSYEVPNGTENQSPRPVTSLGFVNETFFEEKPKAPLPLGRFYIHLNSILNISISE
VHSPIKIIVNTPTQNMQLPWQAVNGNNRLDHDFAFHVDDNFKVSFMFLDIPIEDKSNGSK
GVSATKDVSNGKPAETKSKARKFFDKLFNRRKKRKLNKAAAVENSKAKKSVVIKKVSGTA
TLNLGNVKDSCFGKAFNVEIPIISRGFLEAIPVKINSIGKRTLGNLTLTCLYIPELSVPE
QELPFTLEQATMDLRHVRSNYLYNEGYLYRLEDSSIRRRFVVLRSKQLNFYAEKGGQYLD
TFQLSKTVVSIPMVNFSEAVSNLGLVAGILATSVDRRHVQLFADSKKVCQKWLQVMNSRS
FALDRGTEKLWLQEYVNFMA

>sp|Q9BZB8|CPEB1_HUMAN Cytoplasmic polyadenylation element-binding protein 1 OS=Homo sapiens OX=9606 GN=CPEB1 PE=1 SV=1 ***

MALSLEEEAGRIKDCWDNQEAPALSTCSNANIFRRINAILDNSLDFSRVCTTPINRGIHD
HLPDFQDSEETVTSRMLFPTSAQESSRGLPDANDLCLGLQSLSLTGWDRPWSTQDSDSSA
QSSTHSVLSMLHNPLGNVLGKPPLSFLPLDPLGSDLVDKFPAPSVRGSRLDTRPILDSRS
SSPSDSDTSGFSSGSDHLSDLISSLRISPPLPFLSLSGGGPRDPLKMGVGSRMDQEQAAL
AAVTPSPTSASKRWPGASVWPSWDLLEAPKDPFSIEREARLHRQAAAVNEATCTWSGQLP
PRNYKNPIYSCKVFLGGVPWDITEAGLVNTFRVFGSLSVEWPGKDGKHPRCPPKGNMPKG
YVYLVFELEKSVRSLLQACSHDPLSPDGLSEYYFKMSSRRMRCKEVQVIPWVLADSNFVR
SPSQRLDPSRTVFVGALHGMLNAEALAAILNDLFGGVVYAGIDTDKHKYPIGSGRVTFNN
QRSYLKAVSAAFVEIKTTKFTKKVQIDPYLEDSLCHICSSQPGPFFCRDQVCFKYFCRSC
WHWRHSMEGLRHHSPLMRNQKNRDSS
