# Supplementary material for: Sequence-Based Prediction of Protein Phase Separation: The Role of Beta-Pairing Propensity
Source: Biomolecules. 2022 Nov 28;12(12):1771. doi: 10.3390/biom12121771 (PMC9775558; doi:10.3390/biom12121771)
Supplement: Supplementary file 1 [file biomolecules-12-01771-s001.zip › LLPS_SI.pdf]

Sequence-based prediction of protein phase separation: the role of beta-pairing propensity

Pratik Mullick[1,2,3], Antonio Trovato[1,4*]

1 Department of Physics and Astronomy ‘G. Galilei’, University of Padova, Padova, Italy

2 University of Rennes, INRIA, CNRS, IRISA, Rennes, France

3 Department of Operations Research and Business Intelligence, Wrocław University of Science and Technology, Wrocław, Poland

4 National Institute of Nuclear Physics (INFN), Padova Section, Padova PD, Italy

* antonio.trovato@unipd.it

Supporting Figures and Tables

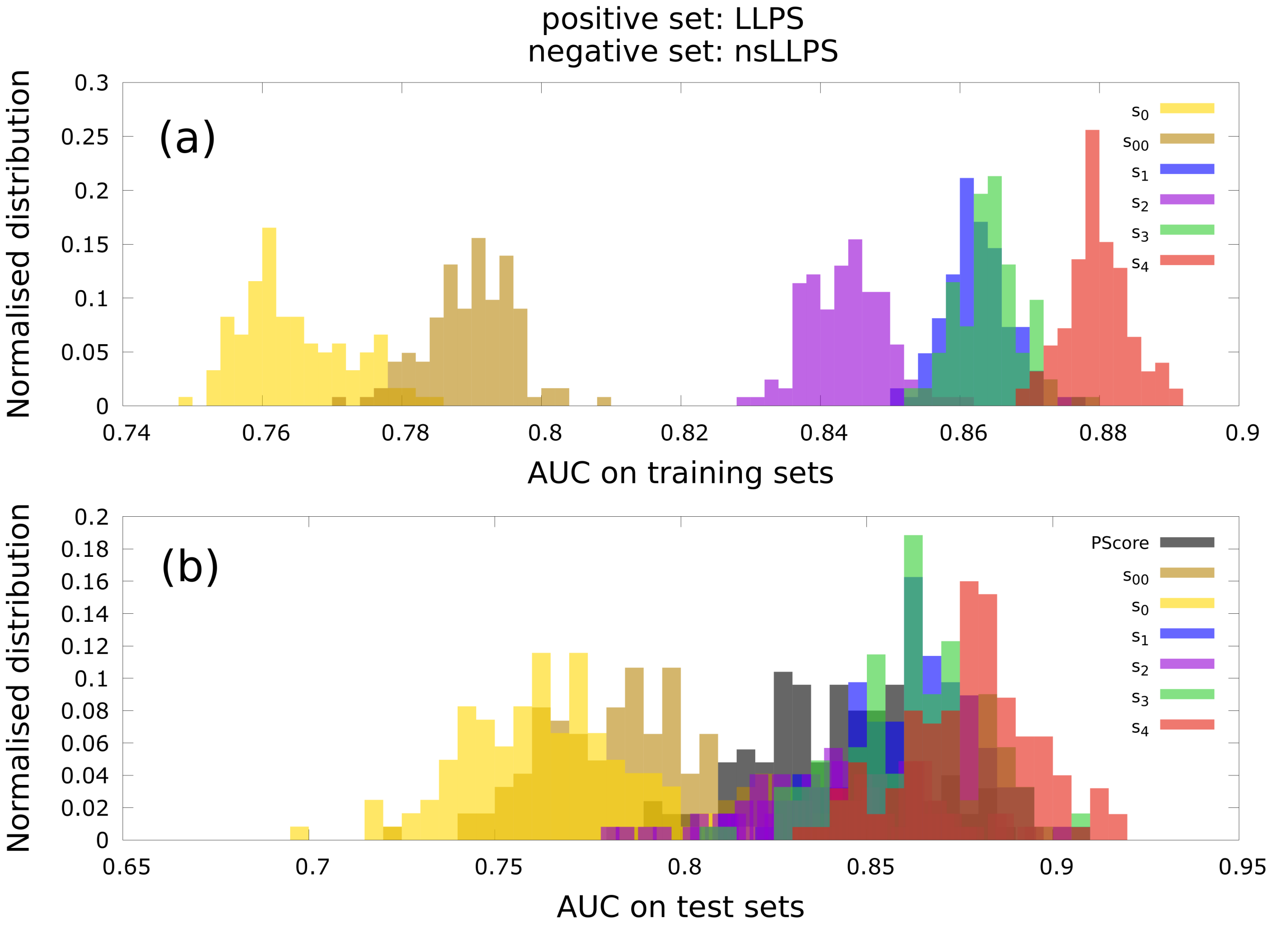

Figure S1. Normalised distributions for the values of AUC on the (a) training set and the (b) test set as obtained from the cross validation procedure. LLPS is the positive set and nsLLPS is the negative set. The AUC values for PScore shown here were obtained on the test sets of cross validation.

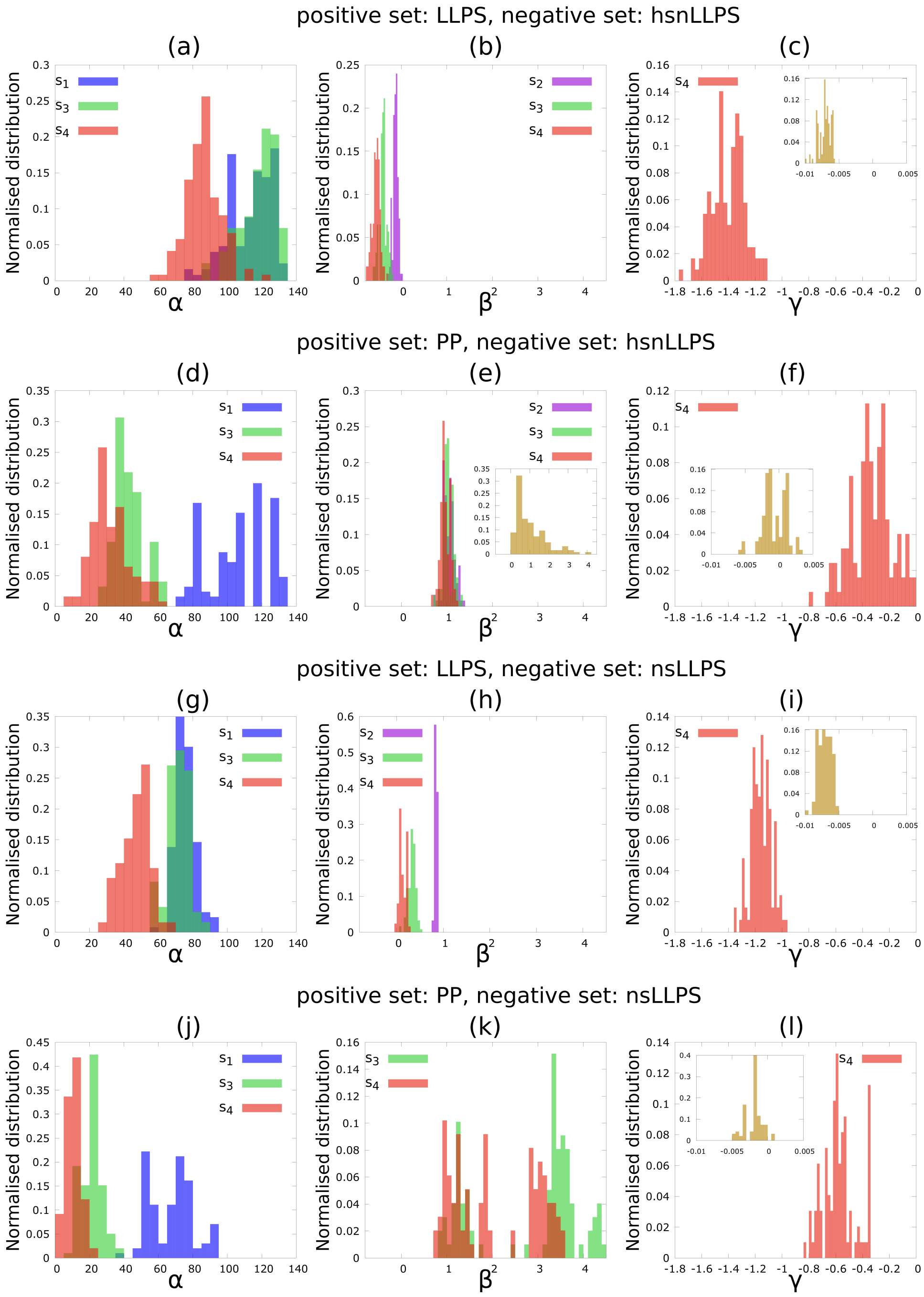

Figure S2. Normalised distributions of the optimised parameters for the different scores. First column: α; second column: β; third column: γ. The normalized distributions for the $\tilde{\beta}$ and $\tilde{\gamma}$ parameters used in the s_0 and s_{00} scores are shown in the insets. First row: LLPS positive set, hsnLLPS negative set; second row: PP positive set, hsnLLPS negative set; third row: LLPS positive set, nsLLPS negative set; fourth row: PP positive set, nsLLPS negative set.

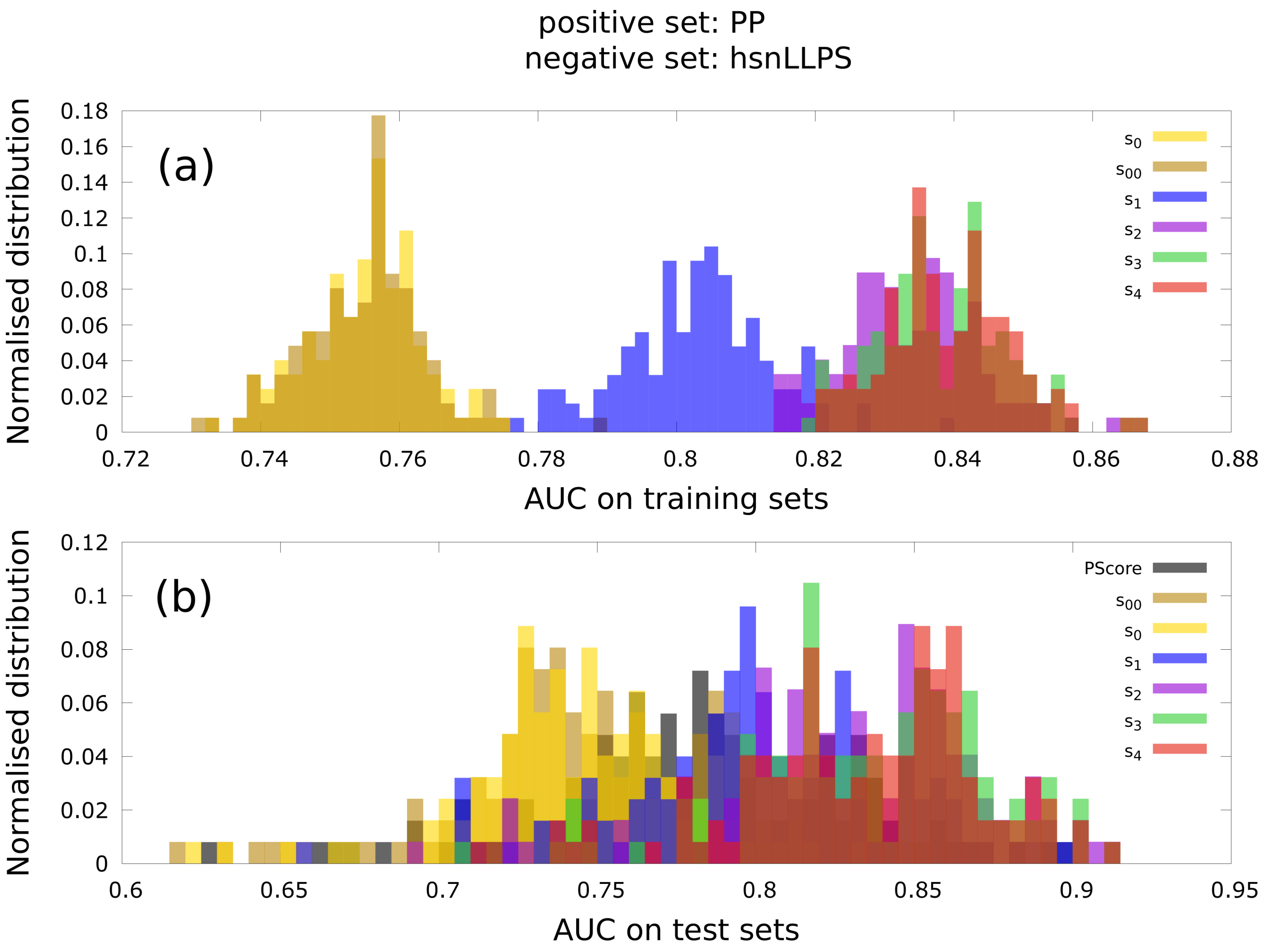

Figure S3. Normalised distributions for the values of AUC on the (a) training set and the (b) test set as obtained from the cross validation procedure. PP is the positive set and hsnLLPS is the negative set. The AUC values for PScore shown here were obtained on the test sets of cross validation.

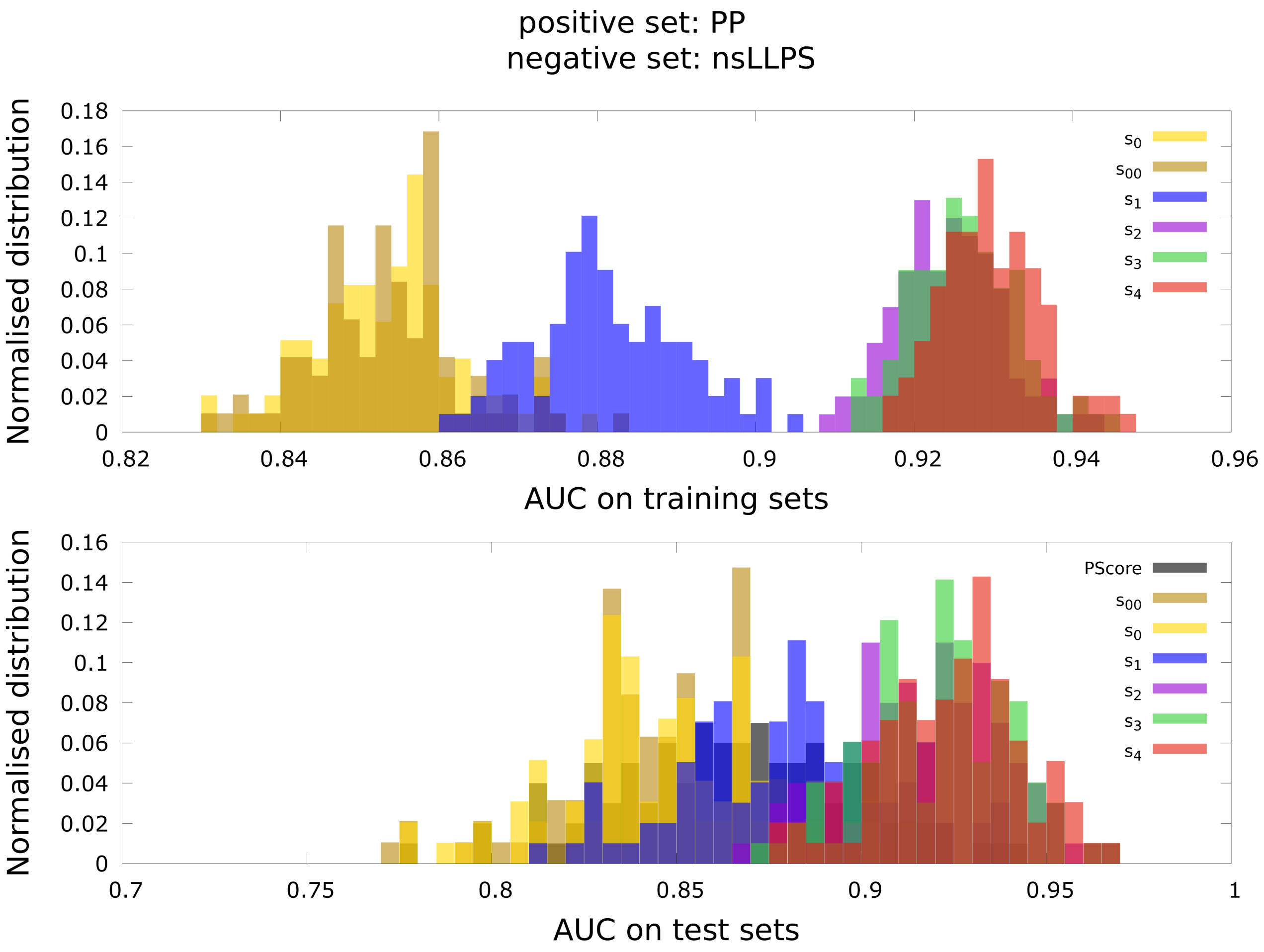

Figure S4. Normalised distributions for the values of AUC on the (a) training set and the (b) test set as obtained from the cross validation procedure. PP is the positive set and nsLLPS is the negative set. The AUC values for PScore shown here were obtained on the test sets of cross validation.

**Scores tested**	$\boldsymbol{F}$	$\boldsymbol{p}$	$\boldsymbol{\eta^2}$
s_0, s_{00}	$F(1, 241) = 64.54$	$< 10^{-6}$	0.211
Pscore, s_1, s_2	$F(2, 368) = 39.06$	$< 10^{-6}$	0.175
Pscore, s_2	$F(1, 246) = 1.623$	0.204	0.006
s_1, s_3	$F(1, 243) = 0.15$	0.699	$\sim 10^{-4}$
s_1, s_3, s_4	$F(2, 367 = 29.84$	$< 10^{-6}$	0.14
s_2, s_3, s_4	$F(2, 367) = 92.75$	$< 10^{-6}$	0.336

Table S1. Results of one-way ANOVAs to check statistical significance between AUC values on the test set for different groups of scoring functions, with LLPS as the positive set and nsLLPS as the negative set.

**Score**	**AUC**	**MCC**	$\boldsymbol{\alpha}$	$\boldsymbol{\beta}$	$\boldsymbol{\gamma}$
PScore	0.84 ± 0.02	0.51 ± 0.04			
s_0	0.76 ± 0.02	0.29 ± 0.03		0.8 ± 18.8	
s_{00}	0.79 ± 0.02	0.32 ± 0.04		0.002 ± 0.032	-0.007 ± 0.001
s_1	0.86 ± 0.02	0.54 ± 0.04	75 ± 6		
s_2	0.84 ± 0.02	0.52 ± 0.04		0.34 ± 0.06	
s_3	0.86 ± 0.02	0.54 ± 0.04	72 ± 6	0.33 ± 0.08	
s_4	0.88 ± 0.02	0.56 ± 0.04	47 ± 8	0.13 ± 0.07	-1.16 ± 0.08

Table S2. Typical values of AUC and MCC on the test set using LLPS as the positive set and nsLLPS as the negative set. The optimised parameters α, β and γ are also summarised here ($\tilde{\beta}$ and $\tilde{\gamma}$ are actually reported for scores s_0 and s_{00}). The numerical entries are the mean or the median (depending on whether the quantity follows a normal distribution or not, respectively, see File S1) $\pm$ the standard deviation of the corresponding quantity.

**Scores tested**	$\boldsymbol{F}$	$\boldsymbol{p}$	$\boldsymbol{\eta^2}$
s_0, s_{00}	$F(1, 190) = 0.334$	0.564	0.002
Pscore, s_1, s_2	$F(2, 296) = 101.2$	$< 10^{-6}$	0.406
Pscore, s_1	$F(1, 197) = 12.41$	5×10^{-4}	0.059
s_1, s_3, s_4	$F(2, 293) = 114.6$	$< 10^{-6}$	0.439
s_2, s_3, s_4	$F(2, 294) = 1.903$	0.151	0.012

Table S3. Results of one-way ANOVAs to check statistical significance between AUC values on the test set for different groups of scoring functions, with PP as the positive set and nsLLPS as the negative set.

**Score**	**AUC**	**MCC**	$\boldsymbol{\alpha}$	$\boldsymbol{\beta}$	γ
PScore	0.87 ± 0.03	0.46 ± 0.06			
s_0	0.85 ± 0.03	0.34 ± 0.06		0.4 ± 4.0	
s_{00}	0.85 ± 0.03	0.34 ± 0.06		1 ± 3	-0.001 ± 0.001
s_1	0.88 ± 0.03	0.48 ± 0.05	67 ± 13		
s_2	0.91 ± 0.02	0.48 ± 0.06		3 ± 1	
s_3	0.92 ± 0.02	0.49 ± 0.07	21 ± 6	3.4 ± 1.2	
s_4	0.92 ± 0.02	0.50 ± 0.06	10 ± 4	1.8 ± 0.9	-0.58 ± 0.12

Table S4. Typical values of AUC and MCC on the test set using PP as the positive set and nsLLPS as the negative set. The optimised parameters α, β and γ are also summarised here ($\tilde{\beta}$ and $\tilde{\gamma}$ are actually reported for scores s_0 and s_{00}). The numerical entries are the mean or the median (depending on whether the quantity follows a normal distribution or not, respectively, see File S1)$\pm$ the standard deviation of the corresponding quantity.

	Espritz		IUPred		GlobPlot	
**Sequence set**	**Dis. Frac.**	**Dis. Seg.**	**Dis. Frac.**	**Dis. Seg.**	**Dis. Frac.**	**Dis. Seg.**
nsLLPS (mp neg. set)	0.16	12	0.08	7	0.14	7
hsnLLPS (hp neg. set)	0.33	22	0.25	14	0.22	11
LLPS (positive set)	0.53	38	0.45	20	0.33	16
S_4 (s_4 True Positives)	0.57	42	0.50	21	0.36	18
$\tilde{S}_4$ (s_4 TP $\setminus$ PScore TP)	0.34	21	0.28	12	0.17	10
S_P (PScore True Pos.)	0.60	44	0.52	22	0.38	19
$\tilde{S}_P$ (PScore TP $\setminus$ s_4 TP)	0.49	29	0.40	17	0.30	14

Table S5. Intrinsic disorder predictions for different sequence sets used in this work. We show predictions from ESpritz, set in the NMR prediction type with a FPR = 0.3 decision threshold, from IUPred-long, and from GlobPlot. Predictor settings are the ones implemented in MobiDB-lite. For each predictor, we report the total disorder fraction (**Dis. Frac.**) and the average length of the predicted disordered segments (**Dis. Seg.**). The sequence sets $\tilde{S}_4$, $\tilde{S}_P$, S_4, S_P were defined in the main text, such that $\tilde{S}_4 \subset S_4$, $\tilde{S}_4 \cap S_P = \emptyset$, $\tilde{S}_P \subset S_P$, $\tilde{S}_P \cap S_4 = \emptyset$. hp = human proteome; mp = mixed proteomes; TP = true positives.
